# Genes Left Behind: Climate Change Threatens Cryptic Genetic Diversity in the Canopy-Forming Seaweed *Bifurcaria bifurcata*

**DOI:** 10.1371/journal.pone.0131530

**Published:** 2015-07-15

**Authors:** João Neiva, Jorge Assis, Nelson C. Coelho, Francisco Fernandes, Gareth A. Pearson, Ester A. Serrão

**Affiliations:** Centro de Ciências do Mar da Universidade do Algarve, Faro, Portugal; University of Connecticut, UNITED STATES

## Abstract

The global redistribution of biodiversity will intensify in the coming decades of climate change, making projections of species range shifts and of associated genetic losses important components of conservation planning. Highly-structured marine species, notably brown seaweeds, often harbor unique genetic variation at warmer low-latitude rear edges and thus are of particular concern. Here, a combination of Ecological Niche Models (ENMs) and molecular data is used to forecast the potential near-future impacts of climate change for a warm-temperate, canopy forming seaweed, *Bifurcaria bifurcata*. ENMs for *B*. *bifurcata* were developed using marine and terrestrial climatic variables, and its range projected for 2040-50 and 2090-2100 under two greenhouse emission scenarios. Geographical patterns of genetic diversity were assessed by screening 18 populations spawning the entire distribution for two organelle genes and 6 microsatellite markers. The southern limit of *B*. *bifurcata* was predicted to shift northwards to central Morocco by the mid-century. By 2090-2100, depending on the emission scenario, it could either retreat further north to western Iberia or be relocated back to Western Sahara. At the opposing margin, *B*. *bifurcata* was predicted to expand its range to Scotland or even Norway. Microsatellite diversity and endemism were highest in Morocco, where a unique and very restricted lineage was also identified. Our results imply that *B*. *bifurcata* will maintain a relatively broad latitudinal distribution. Although its persistence is not threatened, the predicted extirpation of a unique southern lineage or even the entire Moroccan diversity hotspot will erase a rich evolutionary legacy and shrink global diversity to current (low) European levels. NW Africa and similarly understudied southern regions should receive added attention if expected range changes and diversity loss of warm-temperate species is not to occur unnoticed.

## Introduction

Global warming constitutes, together with land-use change and pollution, a major global anthropogenic threat to biodiversity in both marine and terrestrial systems [1–3]. Temperature rise is being accompanied by a cascade of effects, ranging from changes in regional precipitation regimes to seawater chemistry [4,5]. There is a general concern that, for most species, evolutionary responses (e.g. evolution of climatic niches) will be largely outpaced by the observed rate of climatic change, forcing populations to move, acclimatize or perish when facing these new climatic environments. Regionally, climate change has already affected a multitude of species across a diverse range of taxonomic groups, biomes and latitudes. Impacts include local shifts in phenology, physiology and demography, and, perhaps the best documented, shifts in species ranges [6–8].

Range shifts involve regional extirpations and/or colonization of newly suitable areas, often but not restricted to peripheral range edges, as species track their climatic niches [9]. Ecological Niche Models (ENMs) have been increasingly used to forecast such responses to climatic change. ENMs relate distributional and environmental data to determine the environmental factors underlying species distributions, and can be used to project the potential distribution of suitable habitat (as a proxy for species distributions) in future climatic scenarios, including the geographic areas where species may be under higher risk of extirpation and those more likely to provide new suitable habitats for colonization [10,11].

The use of genetic assessments extends ENMs by allowing potential losses of cryptic diversity (i.e. phenotypically invisible standing variation) associated with climate change to be predicted [12]. Genetic variation is seldom evenly distributed across species ranges, particularly in low-dispersal species, where low rates and/or range of migration contribute to the emergence and maintenance of spatial genetic structure. For such species, present day genetic structures commonly retain clear signatures of past range dynamics associated with glacial-interglacial cycles [13]. Where species have maintained a long-term regional presence (e.g. southern European peninsulas), populations tend to be genetically more diverse and/or unique than in recently [i.e., after the Last Glacial Maximum (LGM), *ca*. 20000 years BP] colonized areas [14,15]. This pattern is apparent even in species whose post-glacial distribution exceeds the latitudinal range of refugia by several orders of magnitude. The unique diversity of refugial regions reflects their greater age and stability but also the fact that most populations, notably those located behind colonization fronts, don't contribute to post-glacial expansions [16,17].

Global warming is anticipated to trigger similarly diverse demographic responses across species ranges [18], and thus to cause asymmetric changes in the distribution and abundance of different genetic variants [19]. Even in a benign scenario where extirpations at the trailing edge are counteracted by the establishment of new populations at the leading edge, the spatial redistribution and contrasting fate of lineages may result in significant diversity losses, particularly if diversity and/or endemism are concentrated within the vanishing range [20–23]. The existence of phylogeographic lineages and other geographically-structured components of intra-specific diversity is of particular importance for the global ecological plasticity, evolutionary potential and long-term viability of species in a constantly changing world [24,25]. The identification of regions of higher conservation/evolutionary value at higher risk of disappearing is thus an important step to inform monitoring, conservation or mitigation strategies [19].

Rocky-shore organisms, especially the conspicuous structural species, are regarded as early-warning indicators for the impacts of climate change [26,27]. Isotherms at the ocean surface have been migrating at lower or comparable rates than over land, but where high rates of change occur they tend to extend across broader regions, thus impacting a larger proportion of species ranges [28]. Local demographic responses are also complicated by reduced small-scale variability of sea surface temperatures (but see [29]), and many coastal organisms (e.g. light-dependent or strictly intertidal species) lack the scope for depth migration. Recent analyses further show that marine ectotherms tend to fully occupy the extent of latitudes tolerable within their thermal limits, particularly their potential equatorward ranges [30]. Their latitudinally narrow safety margins thus make them particularly sensitive and responsive to climatic perturbations at both their leading and trailing edges [31–34]. Low dispersal, canopy-forming seaweeds often harbor diverse populations or even endemic lineages at their warmer equatorward ranges, making them a particularly vulnerable marine group [23,35–40]. Some have already been reported [23] or predicted [37,39,41] to be regionally extirpated at these peripheral genetic “hotspots”. Here we forecast future range rearrangements associated with predicted climate change and assess its potential impacts for the global genetic diversity of the canopy-forming seaweed *Bifurcaria bifurcata*.

*B*. *bifurcata* R. Ross 1958 (Brown Forking Weed) is a perennial and hermaphroditic brown alga endemic to the warm-temperate eastern Atlantic. It occurs from southern Morocco (27.5° N) to north-western Ireland (55° N) on moderately exposed rocky shores in the mid/low intertidal and in rock-pools. The range of *B*. *bifurcata* has expanded and contracted along the leading English Channel edge in response to decadal-scale variations in climatic conditions [42], and other transient shifts are apparent in the region [43]. In contrast, at its southernmost range in southern Morocco, and possibly along Western Sahara [44], distributional and demographic information is poor and outdated. Here we show that genetic variation in *B*. *bifurcata* is overwhelmingly concentrated along this overlooked southern range. Our models predict a poleward range shift associated with future climate change that is likely to result, depending on greenhouse gas emissions, in the loss of a distinct and spatially restricted phylogeographic lineage located at the current trailing edge, or even in the complete extirpation of the Moroccan diversity hotspot.

## Materials and Methods

### Occurrence and environmental data

**Fig 1:**
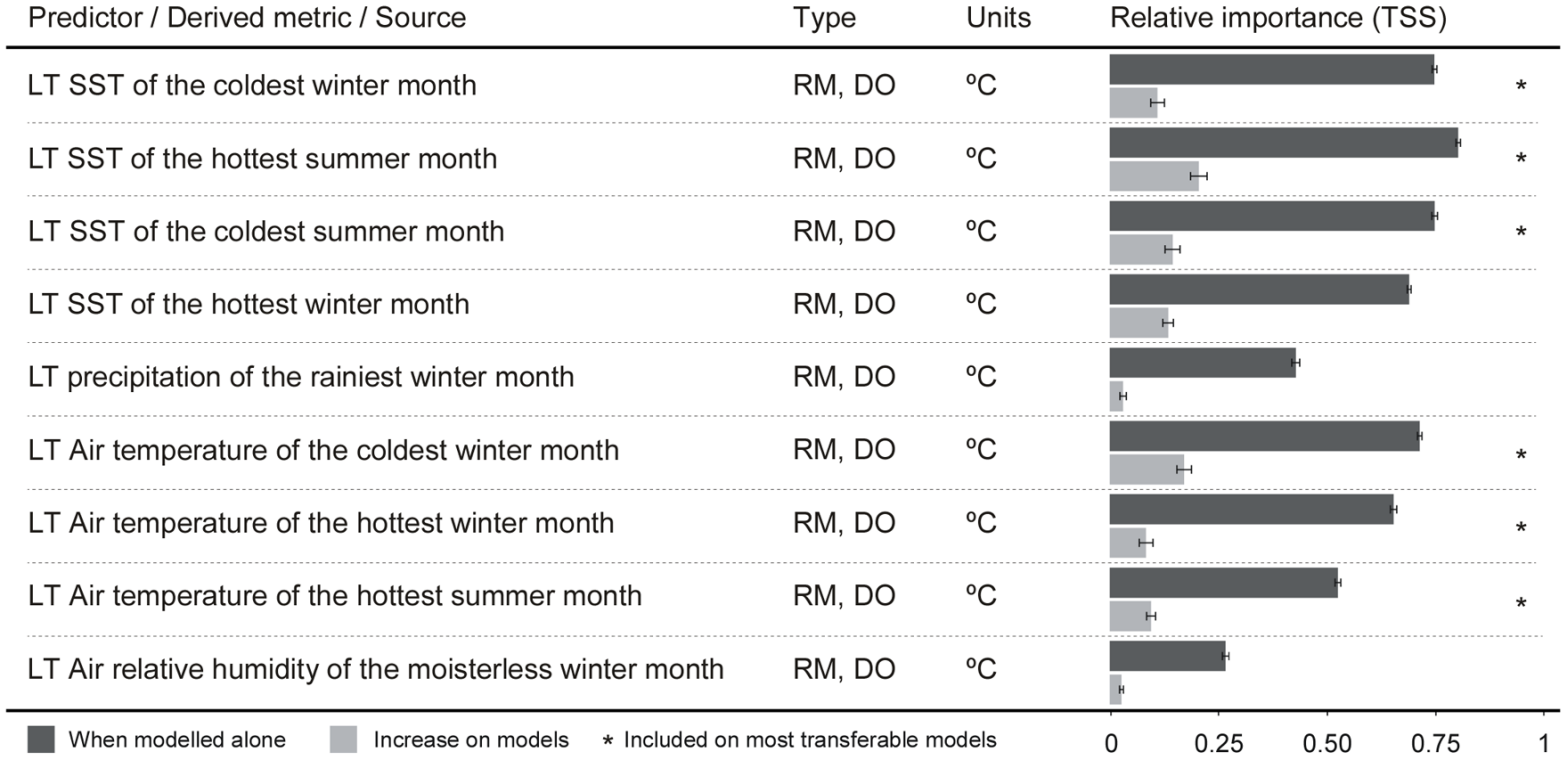
Environmental predictors used to model the Ecological Niche of *Bifurcaria bifurcata*. The relative importance of each environmental predictor (when modelled alone and when added to a model) was assessed using True Skill Statistics (TSS). Asterisks show the predictors included in the ensemble of best transferable models. LT: long-term (1990–2010) monthly mean; RM: remote sensing; DO: direct observation; Resol.: original resolution of data.

Georeferenced occurrences of *B*. *bifurcata* were collated from field observations and the available literature (S1 Table). Records were only considered when described at the shore level. To reduce the effect of spatial autocorrelation, occurrences were gridded to match a 0.1° resolution and multiple entries in a given cell were only considered once. Environmental (climatic) variables relevant for the ecology of intertidal seaweeds were selected for inclusion in the models [35,37]. Raw data were obtained from the Operational Sea Surface Temperature and Sea Ice Analysis (OSTIA; [45]), the European Centre for Medium-Range Weather Forecasts (ECMWF; [46]) and from the high-resolution gridded datasets [47]. Data were summarized as long-term seasonal averages (1990–2010) describing variations in sea surface temperature, air temperature, total precipitation and relative air humidity (Fig 1). Data were gridded to 0.1° resolution using bilinear interpolation to match the distributional data for *B*. *bifurcata*.

### Niche modelling

The niche modelling approach followed an iterative method that identified among possible models those with higher spatial transferability or cross-applicability in cross-validation (i.e., the ability to predict outside the spatial domain of model fitting [10]). Multivariate Adaptive Regression Splines (MARS; [48]) and Boosted Regression Trees (BRT; [49]) were chosen as they have high predictive performance and allow modelling complex, non-linear relationships. Because these require both presence and absence data, a set of pseudo-absences was previously extracted from a habitat suitability surface produced with Mahalanobis distance. In this step, the predictors were normalized to a mean of 0 and a standard deviation of 1 [50]. To avoid over-prediction and to approximate predictions to realized distributions, pseudo-absences were randomly selected from regions where habitat suitability was equal to or lower than 0.2 [51]. Following the recommendation of Barbet-Massin *et al*. [52] for machine learning techniques, equal numbers of pseudo-absences and presences were used.

Cross-validation was performed by randomly dividing the distribution records (presences and pseudo-absences) into two datasets. The first was generated with 70% of the records and was used to train the models with all possible combinations of non-correlated predictors (Spearman’s R < |0.7|; S1 Fig). The remaining 30% were used to test the accuracy of the resulting candidate models. MARS models are dependent on the maximum interaction degree, while BRT models depend on tree complexity, number of trees, learning rate and bag fraction [49]. The optimal parameters for both methods were inferred by performing a 10-fold cross-validation on the training dataset, using the lowest deviance as an estimate of success (e.g. [49,53]). Each combination of predictors was interactively fitted with a range of maximum interaction degrees (1, 2 and 3), tree complexities (1 up to the number of environmental predictors in a given model), number of trees (1000 up to 10000, by steps of 50) and learning rates (0.05, 0.01, 0.005, 0.001). The careful selection of parameters allowed the level of generality of each model to be increased, while avoiding overfitting. A bag fraction of 0.5 was used as it consistently produces good binomial responses [53]. True Skill Statistics (TSS; [54]) were then applied to verify the accuracy of the models, by comparing a predictive map reclassified to maximize the sum of specificity and sensitivity (the capacity of a model to detect true absences and presences, respectively) with the test dataset. TSS values higher than 0.8 indicated an excellent model, between 0.6 to 0.8 a good model and from 0 to 0.6 a null to fair model [54]. The cross-validation design was performed 50 times, with randomly selected training and testing datasets for each, and by selecting a new set of pseudo-absences.

The contribution of each environmental predictor to the models was inferred by determining the mean accuracy (TSS) when a predictor was modeled alone and the mean gain in accuracy when added to a given model. The combinations of predictors with highest potential for transferability per modelling technic (BRT and MARS) were determined by sorting the combinations by decreasing accuracy scores and by running successive Kruskal-Wallis rank tests. In this process, the raw TSS values resulting from the 50 interactions were sequentially added to independent tests until the level of significance (alpha = 0.05) was reached. This allowed pinpointing a threshold in accuracy separating the top-ranked combinations from those retrieving significantly lower accuracy scores.

### Present and future distributions

The full set of distribution records were used to train the final distribution maps. However, because several combinations of predictors could retrieve equally high accuracy scores (on average), the final predictions were produced by ensembling (median function; [55]) the resulting surfaces of the most transferable models for both BRT and MARS. This way, the probability shown in predictions stands for the consensus of multiple single models generated with the two modelling techniques. The ensemble for the present was based on remote sensing data (Fig 1), while those for future times fed on data derived from two Atmosphere-Ocean General Circulation Models (AOGCMs) known to cover most of the variability found between climatic models [56]: the Community Climate System Model (CCSM), and the Model for Interdisciplinary Research on Climate (MIROC). Projections were made for the decades 2040–2050 and 2090–2100 under two extreme scenarios of Representative Concentration Pathway (RCP; [57]). The first considered a substantial reduction of greenhouse gas emissions over time (RCP2.6), whereas the second and more pessimistic contemplated a sustained increase in emission of greenhouse gases (RCP8.5). To evaluate the potential of AOGCMs to predict this species’ distribution, we further produced an ensemble for present times (1990–2010) with data derived from such simulations and tested its accuracy against the distribution records using TSS.

All modeling analyses were performed in R (R Development Core Team 2014; http://www.r-project.org/) using the packages: adehabitat, dismo, gbm, gstat, mda, parallel, raster and SDMTools.

### Sampling and molecular analyses

Individuals of *Bifurcaria bifurcata* (N = 432) were sampled from 18 locations covering the entire known distribution of the species (Table 1). No specific permits were necessary as this species is not endangered or legally protected, and all sampling sites had free public access and were not protected or privately owned. Collection of ca. 4 cm tissue samples did not kill or significantly reduce the biomass of individuals. Samples were collected along *ca*. 100m linear transects. Genomic DNA was extracted from silica-dried tissue using the Nucleospin 96 Plant II kit (Macherey-Nagel, Germany).

**Table 1:** Genetic diversity within sampling sites and selected geographical regions. Indices of diversity are reported for microsatellites and sequence markers. *A*: mean allelic richness; *A*’: number of private alleles; *H*_E_: Nei’s gene diversity; *H*_o_: observed heterozygosity; *F*_IS_: multi-locus inbreeding coefficient (* if significant). Haplotypes frequencies are listed for each population and coded as in Fig 3; *H*: haplotype diversity; π: nucleotide diversity.

Region			Microsatellites (N≈ 24)					mt-cox3 (N≈ 8)			cp-rbc (N≈ 8)		
Site, Country	Code	Latitude, Longitude	*A*	*A*’	*H*_E_	*H*_O_	*F*_IS_	Haplotypes	*H* (10^−3^)	π (10^−5^)	Haplotypes	*H* (10^−3^)	π (10^−5^)
**S Morocco**			**4.33**	**8**	**0.425**			**A1, A2**	**133**	**23**	**A1**	-	-
Tarfaya, MA	TAR	27° 55'N, 12° 57'W	3.00		0.410	0.113	0.730*	A1(7), A2(1)	286	49	A1(6)	-	-
El Ouatia, MA	OUA	28° 24'N, 11° 24'W	2.33		0.330	0.208	0.375*	A1(8)	-	-	A1(8)	-	-
**C Morocco**			**4.17**	**8**	**0.415**			**B1**	-	-	**B1,B2,B6**	**554**	**126**
Essaouira, MA	ESS	31° 30'N, 9° 46'W	2.50		0.320	0.258	0.198*	B1(8)	-	-	B1(7), B6(1)	250	93
El Beddouza, MA	BDO	32° 32'N, 9° 16'W	2.67		0.269	0.222	0.178*	B1(7)	-	-	B1(4), B2(4)	571	107
El Jadida, MA	JAD	33° 12'N, 8° 35'W	3.50		0.348	0.239	0.318*	B1(8)	-	-	B1(2), B2(6)	429	80
**Iberia**			**2.83**	**2**	**0.243**			**B1.B2**	**424**	**72**	**B1,B2,B3,B4,B5**	**558**	**121**
Odeceixe, PT	ODE	37° 26'N, 8° 47'W	1.67		0.188	0.080	0.582*	B1(8)	-	-	B1(8)	-	-
Ribeira de Ilhas, PT	RIH	38° 59'N, 9° 25'W	2.00		0.200	0.181	0.098	B1(3), B2(5)	536	91	B1(8)	-	-
Viana do Castelo, PT	VIA	41° 41'N, 8° 51'W	1.67		0.136	0.080	0.418*	B1(8)	-	-	B1(5), B4(3)	536	10
Lires, ES	LIR	43° 00'N, 9° 15'W	1.17		0.085	0.044	0.494*	B2(8)	-	-	B2(8)	-	-
A Coruña, ES	RCO	43° 22'N, 8° 20'W	1.83		0.140	0.063	0.560*	B1(8)	-	-	B1(8)	-	-
Porcia, ES	POR	43° 33'N, 6° 52'W	1.67		0.163	0.146	0.107	B1(2), B2(6)	429	73	B3(6), B4(2)	429	16
Lastres, ES	LAS	43° 30'N, 5° 16'W	2.00		0.256	0.134	0.481*	B1(7)	-	-	B1(4), B5(3)	571	106
Zumaya, ES	ZUM	43° 17'N, 2° 16'W	1.33		0.046	0.036	0.214	B1(8)	-	-	B1(7)	-	-
**Brittany & British Isles**			**2.67**	**0**	**0.246**			**B1**	-	-	**B1,B5**	**193**	**36**
Piriac-sur-Mer, FR	PSM	47° 22'N, 2° 33'W	2.17		0.192	0.132	0.316*	B1(7)	-	-	B1(8)	-	-
Saint-Briac-sur-Mer, FR	SBM	48° 38'N, 2° 08'W	1.33		0.020	0.007	0.662*	B1(7)	-	-	B1(8)	-	-
Plymouth, UK	PLY	50° 18'N, 4° 05'W	1.33		0.088	0.014	0.846*	B1(8)	-	-	B1(7)	-	-
Clonakilty, IE	COR	51° 35'N, 8° 47'W	1.50		0.122	0.056	0.550*	B1(8)	-	-	B1(7)	-	-
Galway, IE	GAL	53° 08'N, 9° 13'W	1.17		0.081	0.058	0.290	B1(7)	-	-	B1(4), B5(4)	571	106
**Morocco (npop = 5)**			**6.33**	**18**	**0.554**			**A1, A2, B1**	**505**	**257**	**A1, B1, B2, B6**	**696**	**288**
**Europe (npop = 13)**			**3.67**	**2**	**0.265**			**B1, B2**	**302**	**51**	**B1,B2,B3,B4,B5**	**439**	**91**

A geographically comprehensive panel of individuals was used to assess the polymorphism of 25 microsatellite loci identified *in silico* by screening with MSATCOMMANDER [58] an EST library generated from heat-shocked tissue. Six loci were found to be polymorphic and to give consistent patterns of amplification and size scoring, and these were used to produce multi-locus genotypes for all individuals (see S2 Table for primer sequences and amplification details). In addition, 8 individuals from each population were sequenced for fragments of the mitochondrial gene *cox3* (618bp) and the chloroplast *rbc* operon (527–537 bp), the latter spanning the intergenic spacer between the large (*rbc*L) and small (*rbc*S) sub-unit genes. Primer sequences were developed using published sequences available in Genbank database (*cox3*: EU681436 [59]; *rbc*L: AY590500 [60]; *rbc*S: FM957154 [61] and AF132472 [62]). Amplified fragments were run in an ABI PRISM 3130xl automated capillary sequencer (Applied Biosystems) at CCMAR, Portugal. Microsatellite alleles were manually scored in STRAND (Veterinary Genetics Laboratory, University of California, Davis; http://www.vgl.ucdavis.edu/STRand) using the 500 LIZ size standard (Applied Biosystems). Sequences were aligned, proofread and edited in GENEIOUS 4.8 (Biomatters; http://www.geneious.com).

### Genetic data analyses

Summary statistics of microsatellite genetic diversity were calculated with GENETIX 4.05 (Laboratoire Génome, Populations, Interactions, Université de Montpellier II; http://kimura.univ-montp2.fr/genetix), including allele frequencies, mean allelic richness (*A*), Nei’s gene diversity (*H*_E_), observed heterozygosity (*H*_O_) and inbreeding coefficients (*F*_IS_). Pairwise *F*_ST_ and *D* (*D*_est_; [63]) were estimated with SMOGD 1.25 [64]. Genetic structure was visually inspected using a factorial correspondence analysis (FCA) implemented in GENETIX. A neighbor-joining (NJ) population tree based on allele frequency data was constructed with POPTREE [65] using Nei’s *D*_A_ distances [66]. A Bayesian, model-based genetic admixture analysis implemented in STRUCTURE 2.3 (Pritchard Lab, Stanford University; http://pritchardlab.stanford.edu/structure.html) was run with all individuals combined and with no prior population assignment. Each number of assumed populations (K, set sequentially from 1 to 12) was run five times using a burn-in of 200000 iterations and a run-length of 1000000 iterations. The “best” number of genetic clusters was inferred following the ΔK choice criterion of [67].

The geographic distribution of the *cox*3 and *rbc* haplotypes was mapped and their genealogic relationships were inferred using a median-joining algorithm implemented in Network 4.6 (Fluxus-Engineering; http://www.fluxus-engineering.com/sharenet.htm). Haplotype and nucleotide diversities were calculated for each population and selected geographical regions using DNASP 5.10 [68].

## Results

### ENMs and range projections

The contribution of each environmental predictor to the models was very similar between MARS and BRT (S3 Table). For brevity, only the values obtained with BRT are thus discussed. The global distribution of *Bifurcaria bifurcata* was mostly explained by air and sea surface temperatures (Fig 1). All air and sea surface predictors had good accuracies when modeled alone (with the exception of summer air temperatures). When combined with other predictors, they allowed an increase in the mean TSS from 0.11±0.01 up to 0.19±0.01. When modelled alone, precipitation, relative humidity and hottest summer air temperatures retrieved null to fair models (accuracies ranging from 0.29±0.01 to 0.52±0.01). These predictors also provided little contribution to the accuracy of the models when modelled with other predictors (gains in TSS from 0.02±0.01 to 0.09±0.01).

**Fig 2:**
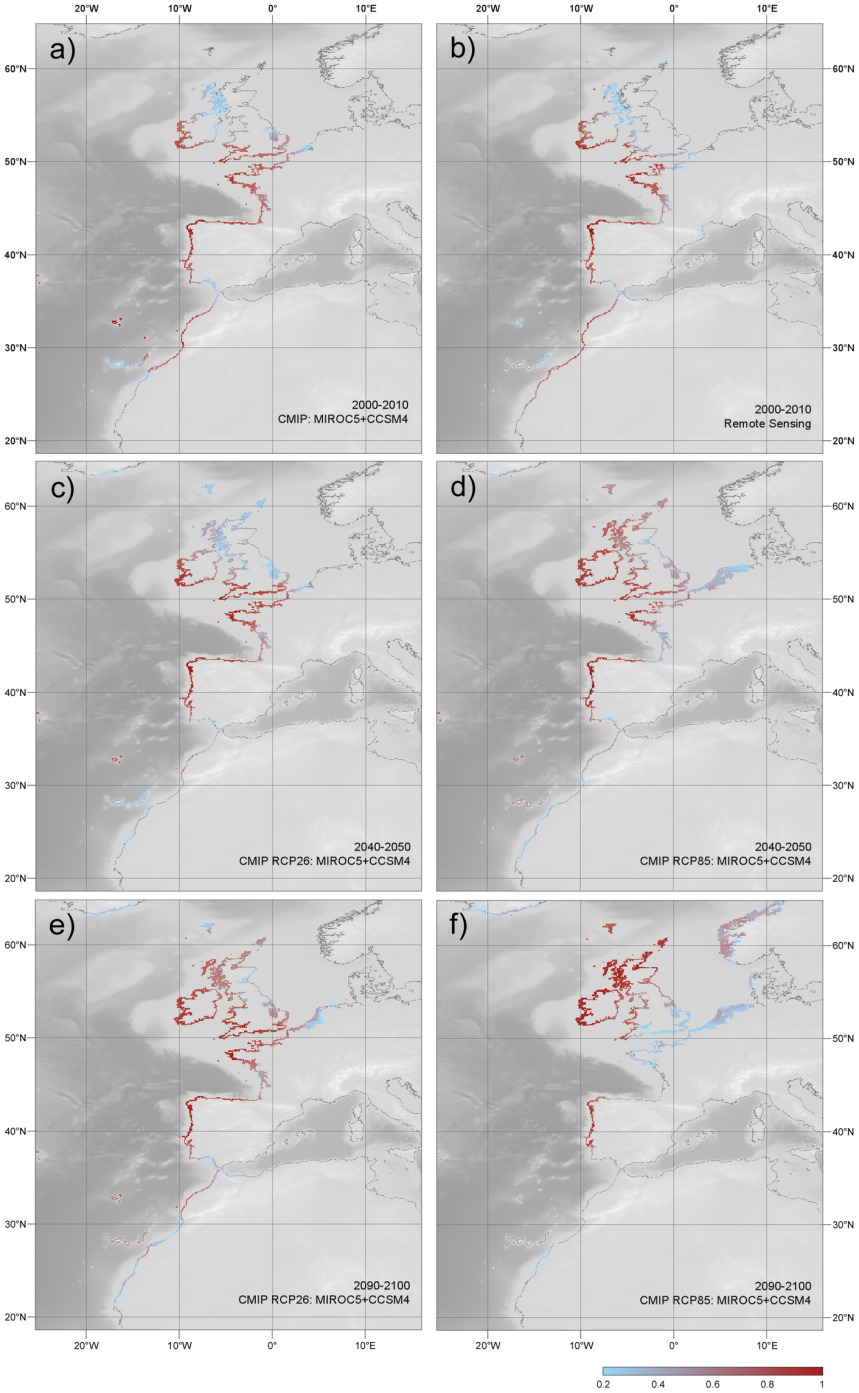
Potential (modelled) distribution of *Bifurcaria bifurcata* in the present and in the near-future. Maps depict the estimated probability of occurrence (as a function of habitat suitability) determined by ensembling multiple MARS and BRT models for the present using **a**) circulation models, and **b**) remote sensing data; and near-future range projections for 2040–2050 (**c**,**d**) and 2090–2100 (**e**,**f**), according to the RCP2.6 (**c**, **e**) and RCP8.5 (**d**, **f**) emission scenarios.

The ensemble model for the present using remote sensing data had an excellent performance (TSS: 0.98). This and the ensemble based on the AOGCMs showed that presently the niche of *B*. *bifurcata* is theoretically distributed from Western Sahara to western Ireland, with some discontinuities in the Gulf of Cadiz and the Bay of Biscay (Fig 2a and 2b). By 2040–50, currently available habitat throughout Western Sahara and southern Morocco was predicted to be lost in both emission scenarios (Fig 2c and 2d). In the benign RPC2.6 scenario, the potential distribution of *B*. *bifurcata* would still include central Morocco and consolidate in the English Channel (Fig 2c). In the more extreme RPC8.5 scenario, central Morocco was predicted to become largely marginal (0.3<P<0.4), but available habitats would extend further north along the Irish Sea and Atlantic Scotland (Fig 2d). A similar poleward expansion is expected in the 2.6 scenario only by 2090–2100s, accompanied by the improvement of conditions along the Western Sahara (Fig 2e). By comparison, in the RCP8.5 scenario, the southern trailing edge of *B*. *bifurcata* was projected to advance as far north as western Iberia (~38.5° Lat., Fig 2f), and northeastern Iberia, Brittany and the English Channel were also forecast to become largely unsuitable. The loss of these southern range areas was concomitant with the emergence of adequate habitat along the Norwegian coast.

### Organelle phylogeographies

**Fig 3:**
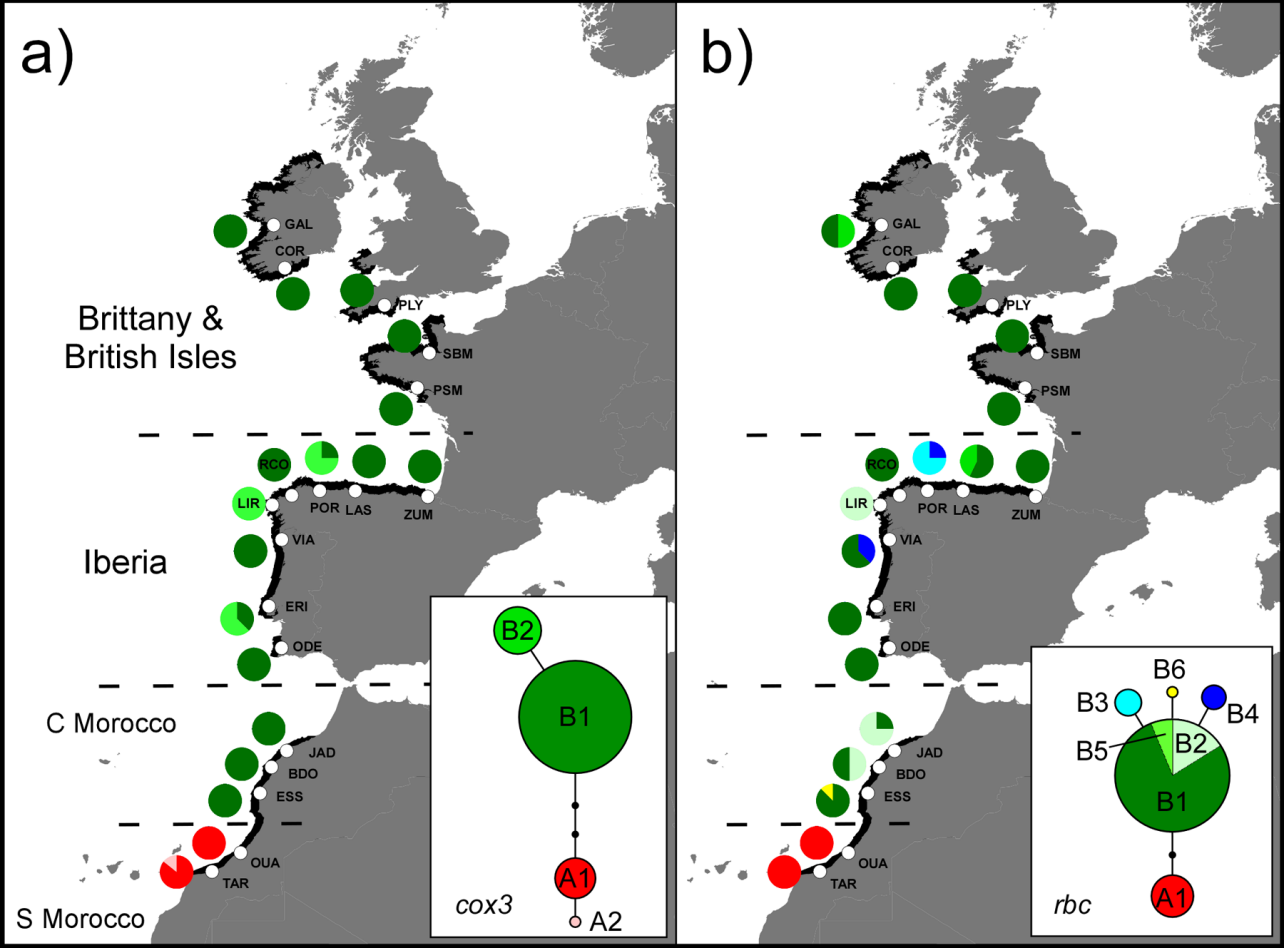
Organelle phylogeographies. Genealogies (insets) and geographic distributions (maps) of **a)** *cox*3 and **b)** *rbc* haplotypes sampled throughout the range (black shoreline) of *Bifurcaria bifurcata*. Pie charts depict haplotype frequencies at each site (white dots, see Table 1 for site codes and haplotype IDs). The horizontal dashed lines delimit the geographical subdivisions considered. In the networks sampled haplotypes are represented by circles sized to their global frequency, links represent a single nucleotide change and black dots represent inferred, unsampled haplotypes. *Rbc* haplotypes B1, B2 and B5 differ in a pentanucleotide repeat.

A total of 4 *cox*3 (Genbank accession nos. KR002845-48) and 7 *rbc* (accession nos. KR002849-55) haplotypes were identified in the 144 sequenced individuals of *Bifurcaria bifurcata*. Both markers exhibited low haplotypic diversity and sequence divergence, but they were concordant in delimiting two phylogeographic groups (Fig 3). The first, A, comprised the southernmost populations of Tarfaya and El Ouatia, and was separated by three (*cox*3, Fig 3a) or two (*rbc*, Fig 3b) single nucleotide polymorphisms (SNPS) from the second phylogroup, B. The latter, comprising the remaining 16 populations, was more diverse than phylogroup A. Central haplotypes A1 and B1 of both markers were widespread and numerically dominant within their ranges, and were never found mixed. Related haplotypes such as B2 (*cox*3), and B3 and B4 (*rbc*), were found in high frequencies in specific populations of northwest Iberia. Additional diversity was recovered within the *rbc* spacer, where variations in the repeat number of a pentanucleotide microsatellite distinguished the haplotypes B1, B2 and B5 (Fig 3b). These were present in NW Iberia but also in central Morocco (B2) and Ireland (B5). Regionally, *π* was much higher in Morocco (both phylogroups present, *π*_cox3_ = 0.00257; π_rbc_ = 0.00288) than in Europe (*π*_cox3_ = 0.00051; *π*_rbc_ = 0.00091).

### Microsatellite-based population structure

**Fig 4:**
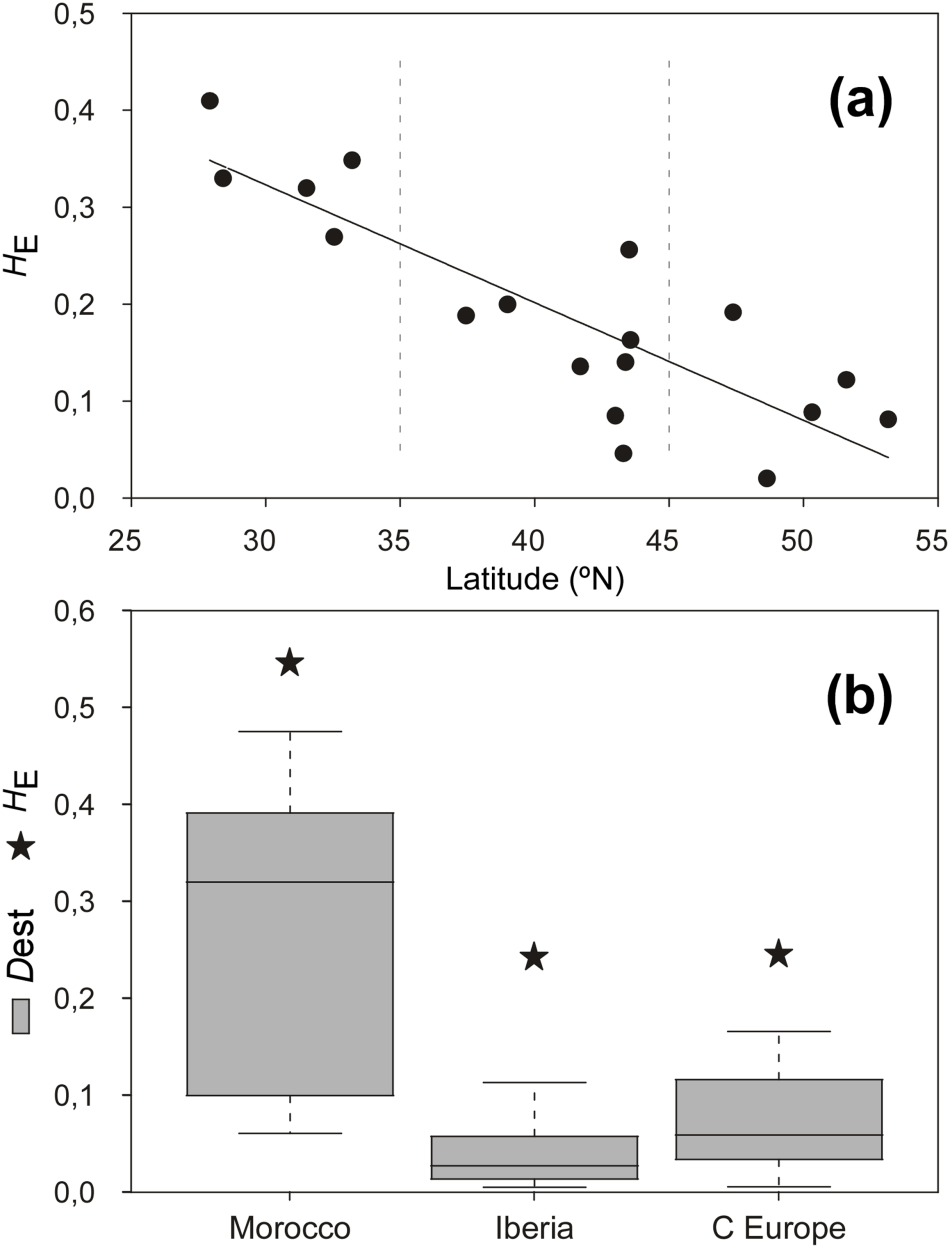
Geographic patterns of (nuclear) genetic diversity and differentiation. **(a)** Nei’s gene diversity (*H*_E_) of populations regressed against latitude. **(b)** Box-plot of *D*_est_ pairwise differentiation of populations and regional *H*_E_ (stars) within Morocco (N = 5), Iberia (N = 8) and Brittany/British Isles (C Europe, N = 5).

**Fig 5:**
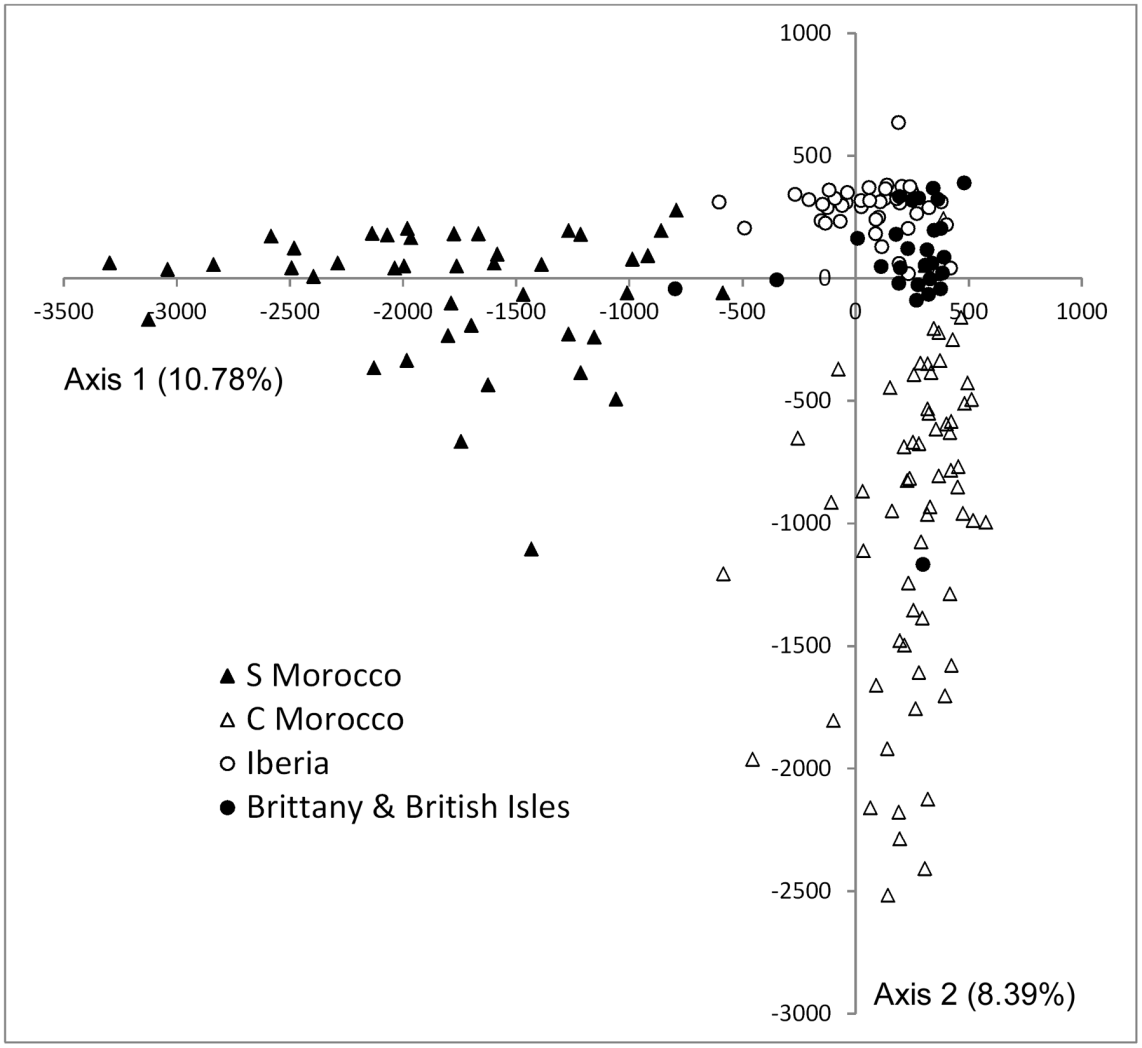
Factorial Correspondence Analysis (FCA) scatter diagram. Individual multilocus genotypes of *Bifurcaria bifurcata* are labelled according to their geographical region of origin.

The 6 microsatellite loci revealed a total of 34 alleles (2–10 per locus). Most populations exhibited heterozygote deficiency (significantly positive *F*_IS_). Within populations, genetic diversity (*A* and *H*_*E*_) was higher throughout Morocco (2.33< *A*< 3.5; 0.269< *H*_E_< 0.410) than in Iberia, Brittany or the British Isles (*A*<2.0; *H*_E_< 0.2), with very few exceptions (e.g. Lastres, Table 1). A decreasing latitudinal trend was apparent for intra-population *H*_E_ (Fig 4a). Globally, the region of Morocco stood out in both levels of genetic diversity and pairwise differentiation among populations (Fig 4b). Within this region, *D*_est_ was highest between pairs of southern (TAR, OUA) and central (ESS, BDO, JAD) populations (S4 Table). The spread of multilocus genotypes in the FCA (Fig 5) illustrates well the higher genetic diversity (and differentiation) of *B*. *bifurcata* in Morocco, and its greater homogeneity elsewhere. It also highlights the distinct genetic compositions of southern and central Moroccan populations.

**Fig 6:**
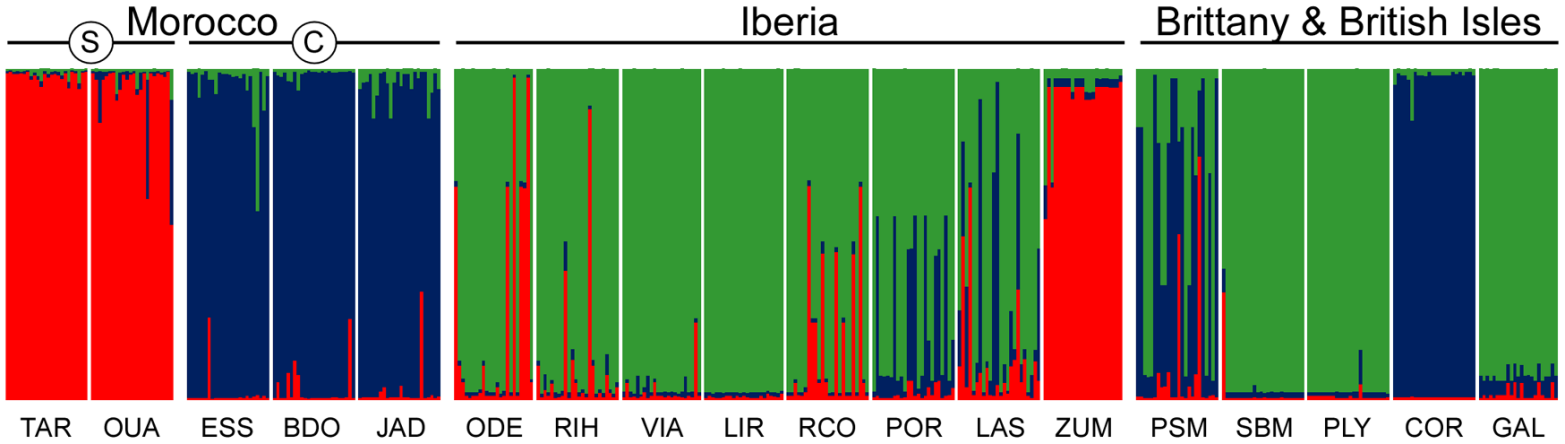
Genetic subdivision based on structure. The proportions of individual multilocus genotypes of *Bifurcaria bifurcata* (vertical bars) assigned to each of the 3 virtual genetic groups are depicted in a different color. Population codes as in Table 1.

**Fig 7:**
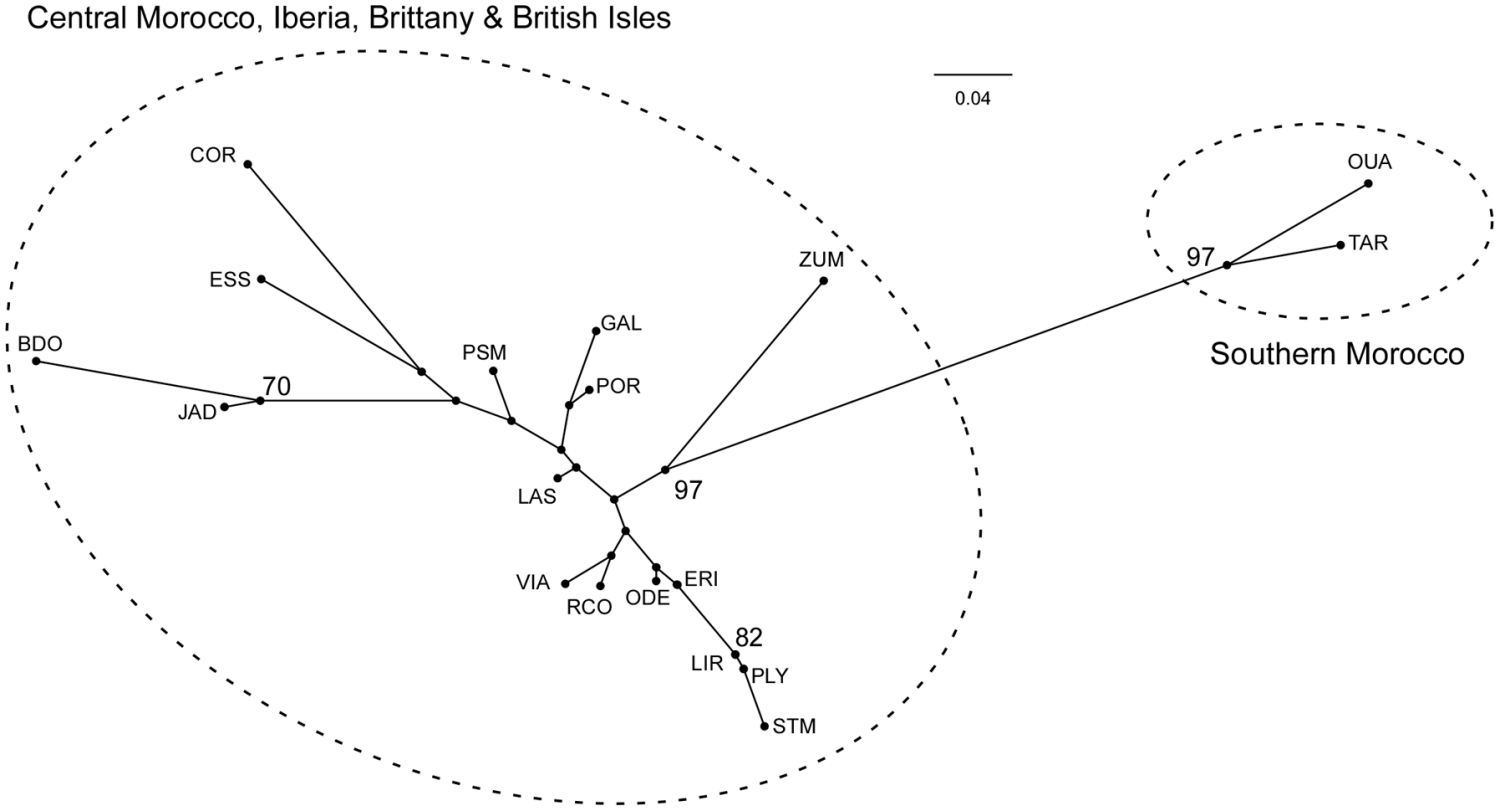
Unrooted NJ tree based on Nei’s *D*_A_ pairwise distances. Circles represent the inferred organelle lineages. Population codes as in Table 1.

*B*. *bifurcata* was subdivided in three major genetic clusters in the STRUCTURE analysis (S2 Fig), which crudely separated southern and central Moroccan from European populations (Fig 6). However, and also apparent in the NJ population tree (Fig 7), one additional European population was largely assigned to either southern (Zumaya) or central (Cork) Moroccan clusters. Further subdivision of genotypes into 9 smaller clusters represented a weaker level of population subdivision with poorer geographic resolution (data not shown).

## Discussion

A wealth of empirical, biogeographical, and niche modelling studies have demonstrated the key importance of marine and terrestrial thermal environments in setting the general distributions (and range shifts) of intertidal sessile species [31,35,69–71]. Predictably, the distribution of *Bifurcaria bifurcata* was also explained by seasonal extremes of air and sea surface (SST) temperatures. The coldest winter temperatures and hottest summer temperatures (both air and SST) should be particularly relevant in setting the northern and southern limits respectively. Predictors nevertheless interact in complex ways, and critical air and sea thresholds do not coincide spatially. At its northern limits in the British Isles, *B*. *bifurcata* is essentially restricted to rock-pools, presumably due to excessively cold air winter temperatures [72] as supported by our results. Similarly, lower SSTs around upwelling regions in southern Morocco help explain its persistence in otherwise seasonally (summer) unfavorable thermal environments.

Our modelling approach, weighting a set of best candidate models, showed excellent transferability and high consistency between the modelled distribution of *B*. *bifurcata* and the observed records. Weighting different sources of data allowed to partially capture the inherent uncertainty of predicting ranges using niche based models [73]. The similar high performances obtained when using environmental data derived from AOGCMs further confirms the advantage of capturing the consensus of multiple models (i.e., ensemble modeling) and shows the ability of these simulations to estimate climate variability at regional scales, at least for present times. Our results predict important near-future range changes for *B*. *bifurcata*, although the course and extent of shifts differed between emission scenarios. By 2040–50, and irrespective of the emission scenario, the severe deterioration of environmental conditions throughout southern Morocco and the Western Sahara was predicted to lead to a northward repositioning of the trailing edge to central Morocco. Local probabilities of occurrence there were nevertheless much lower in the RCP8.5 than in the RCP2.6 scenario, indicating a more marginal and spatially restricted presence in the former.

The forecast southern ranges differed much more significantly between emission scenarios by the end of the century. While the improvement of environmental conditions in the RCP2.6 scenario could allow *B*. *bifurcata* to reoccupy its lost southern Moroccan range, their aggravation in the RCP8.5 scenario predicts the elimination of the species from NW Africa and southwest Iberia. The potential southern limit of *B*. *bifurcata* by 2100 may thus be located either along the coast of the Western Sahara or central Iberia, depending on emission levels. At the leading edge *B*. *bifurcata* was predicted to experience a significant poleward expansion along western Scotland. In the RCP8.5 scenario, it may even reach Norway, although in this case large distributional gaps are predicted in northern Iberia and the English Channel.

Our results show that climate change will affect both the species range and its gene pool. There will be positive and negative population-level effects, dependent on local shifts in conditions across the distribution. Even if range centers and southern edges varied between emission scenarios, globally this species is also predicted to expand northwards and maintain, as presently, a relatively ample latitudinal distribution. In the RCP2.6 scenario, it could even increase its latitudinal span. Assuming that *B*. *bifurcata* will be able to swiftly track its moving niche envelope, the species as a whole is not expected to be facing any significant risk of near-future extinction. However, as shown below, these shifts are likely to have negative impacts on its global genetic diversity. Diversity loss across climatic shifts largely depends on the geographical distribution of genetic diversity. In *B*. *bifurcata*, diversity is rather skewed along the distributional range, concentrated within the vanishing area most at risk and low at its leading expanding edge; the potential biodiversity loss from climate change may thus be severe [74].

*Bifurcaria bifurcata* is composed of two divergent lineages with contrasting distributions and range sizes, as shown by the mitochondrial and chloroplast sequences. Phylogroup A was only detected in a *ca*. 170km stretch of shoreline at its modern rear-edge in southern Morocco (0.5 degrees in latitude), whereas phylogroup B occupied the remaining Moroccan and European distribution (> 20 degrees). Cytoplasmic genomes are inherited maternally in fucoid algae (reviewed in [75]), but their mutations evolve independently. Therefore, their phylogeographic congruence should reflect a truly distinct evolutionary history, and not a stochastic or selective, gene-specific, event. This inference is further reinforced by microsatellite data, which similarly recovered the peripheral populations of Tarfaya and El Ouatia as a unique genetic cluster, most genetically distant from all others. Multilocus genotypes also differentiated central Moroccan from European populations, making Morocco home for two unique genetic groups. Irrespective of the marker used, the Moroccan coast as a whole was the most genetically diverse region, harboring the most diverse and also the most divergent populations. By contrast, European populations were less diverse and differentiated.

It remains difficult, with the data at hand, to establish the historical demographic contexts promoting (and maintaining) the genetic divergence (and exact spatial segregation) of these two main phylogroups. Given their differentiation, the observed pattern may result from the recent secondary contact of former allopatric groups. However, the high genetic diversity found in these groups (relative to the remaining distributional range) supports long term regional persistence of large populations rather than recent expansions, which are often associated with loss of diversity in fucoids [36]. We further hypothesize that the apparent lack of admixture is maintained by inefficient dispersal. This could result partly from the species own reproductive biology; dispersal must be mediated by drifting fragments carrying mature reproductive structures able to release gametes following a drifting period, and even after dispersal priority colonization effects might reduce establishment success [76]. A particularly interesting and novel hypothesis is that, for this particular contact region, the strong upwelling regime is acting as an oceanographic dispersal barrier by causing offshore dispersal of drifting fragments.

The pattern of high and distinct diversity at the lower latitudes is likely to reflect the effects of the last glaciation(s), as the advance of the ice-sheets and permafrost belts eliminated most temperate species from colder, high-latitude areas. The lower levels of genetic diversity and differentiation found in Europe are a common signature of consecutive bottlenecks due to founder events associated with recent (post-glacial), stepping stone colonizations originating in a single or limited set of source populations [17,77]. The phylogeographic data clearly identifies lineage B as the source of the colonization and shows that lineage A, possibly due to its distance from the colonization front, has not participated in the European expansion. Many cold-temperate seaweeds are similarly characterized by largely parapatric lineages at their warmer ranges and a single widespread lineage occupying higher latitudes [35,36,38,78]. However, the warm-temperate *B*. *bifurcata* has its extant diversity hotspots located in northwest Africa, much beyond other known northeast Atlantic (European) seaweed refugia.

It is generally assumed that range dynamics, and particularly population extinctions, will have the highest impact for global patterns of genetic diversity in the next century of climate change. Indeed, given the short evolutionary time frame (<100 years) considered here, the impacts of mutation and lineage sorting will be negligible. Also, at the warm range edge local alleles are more likely to go extinct than to disperse to other populations. Effective gene flow is generally very low in direct-developing structural seaweeds, as they have limited dispersal abilities (in the range of centimeters to dozens of meters for eggs, e.g. [79,80]) and are prone to experience density-barrier effects [38,76]. The high levels of local inbreeding and the existence of steep phylogeographic discontinuities observed in organelle markers confirm this. Finally, genetic diversity in expanding ranges usually represents only a sub-set of that present in (already depleted) source populations due to founder events and genetic surfing, and it is not expected to increase in recolonized areas. The current genetic diversity in populations inferred to persist in the near future was thus considered to represent a good proxy for the global future genetic diversity of *B*. *bifurcata*.

By 2040–2050, the elimination of this species from southern Morocco will predictably result in the extinction of the unique lineage A. Such loss would be irreversible and independent of future range changes. In the CPR2.6 scenario *B*. *bifurcata* may recover its lost southern range by the end of the century, but potential colonizers will likely originate from the nearest surviving sites, which will be central Moroccan populations belonging to lineage B. The CPR8.5 scenario is even more negative. By 2090–2100, *B*. *bifurcata* could be completely eliminated from Morocco, where its presently high diversity and divergence requires large persistent populations, indicating that it has had a relatively stable presence during the past glacial-interglacial cycles. In addition to lineage A, this refugial region harbors the highest regional and intra-population microsatellite diversity, including 18 private alleles (only two in Europe); its loss would shrink the global genetic diversity of *B*. *bifurcata* to the levels presently found in the rather homogenous European genetic cluster. The potential loss of the endemic lineage A is particularly relevant due to its distinctiveness. Individual lineages have partially independent evolutionary trajectories in unique environmental spaces, and thus are also likely to harbor unique and potentially adaptive genetic variability [14,81,82]. The same holds for the diverse, therefore old, central Moroccan populations. Erasing this rich evolutionary legacy and ending the ongoing diversification processes in Morocco will most likely result in reduced functional diversity, ecological resilience and evolutionary potential of the species as a whole.

Estimates of genetic loss, here and elsewhere, depend on the accuracy of the data (range limits, lineage distributions) and of the predictions of range loss. It is possible that the distribution of phylogroup A extends further north along the transitional region between El Ouatia (phylogroup A) and Essaouira (phylogroup B), increasing survival chances (in the best scenario CRP2.6) despite the anticipated extinction of its two presently known populations. The complete extirpation of the entire Moroccan hotspot by the end of the century (CRP8.5) may also be overestimated if some populations unpredictably persist in suitable habitat islands. Local habitat refugia in the warm edge has been documented for another intertidal fucoid alga [83] and *B*. *bifurcata* itself has a local habitat refugium in the northern range where it only occurs in tide pools never exposed to critically cold winter air temperatures.

Meteorological (e.g. cloudiness), oceanographic (e.g. upwelling) or tidal (e.g. time of low tides) eccentricities can also create atypical thermal environments over spatial scales of biogeographic significance, that may regionally superimpose onto latitudinal temperature clines [29,84]. Regular upwelling and the tropicalization of the inner Biscay Bay cause several cold-temperate seaweeds *such as Himanthalia elongata* and *Fucus serratus* to occur in isolated pockets in northwest Iberia [85,86], well beyond their range centers. Kelp forests of *Laminaria ochroleuca* and *Saccorhiza polyschides* also occur in small isolated areas in central Morocco where upwelling is stronger. However, a recent SST-warming trend in Morocco was shown by [23] who noted that the buffering effect of upwelling was insufficient to rescue (estuarine) populations of *Fucus vesiculosus* from extinction. These observations imply that other variables, namely related to the terrestrial climate, are as relevant as oceanographic conditions in setting range limits of southern intertidal species [37,83]. It remains unclear how the northwest African upwelling system will evolve in the coming decades [87,88], but even if strengthened it may be insufficient to create a thermal refugium allowing *B*. *bifurcata* to escape the effects of projected climatic changes.

In the northeast Atlantic, most records of range changes in the shallow marine realm involve limits located along the European coast (e.g. [42,89,90]). This is the case for poleward expansions of warm-temperate species with northern limits in Europe rather than cold temperate species expanding deeper into the Arctic (but see [91]). Likewise, reports of range contractions involve mainly local southern extinctions of cold-temperate species with southern limits in European areas like Brittany and Iberia rather than extinctions of warm-tolerant species from southerner African regions (but see [23]). This bias could be partially attributed to the spatial and seasonal heterogeneity of climate change [92], but must also reflect the scarcity of marine records beyond developed European countries. Species surveys along N Africa (where several warm-temperate species have their southern boundaries) and in the Arctic realm (where cold-temperate species reach their northern limits) are rarer and typically sketchier; as a result, the known distributions and limits of species there are much coarser than throughout their European centers of distribution. Recent or near future range shifts in these understudied regions are thus more likely to be overlooked. Aggravating this problem, many phylogeographical surveys fail to adequately sample remote southern areas, and thus the potential loss of cryptic genetic biodiversity associated with future range changes is also more likely to go unnoticed there.

Our study of a warm-temperate seaweed clearly highlights the problem of survey bias. The distribution of *B*. *bifurcata* in European countries is well known and based on abundant and geographically precise occurrence records (e.g. [93,94]). The comparison of historical and recent surveys, for instance, shows that *B*. *bifurcata* has been expanding and contracting its range in southwest England in response to decadal-scale variations in climatic conditions [42]. There, *B*. *bifurcata* was flagged as an intertidal indicator of climate change (MARCLIM project, [95]). By comparison, the paucity of records available for Morocco prevents the recovery of demographic trends in its southernmost range. *B*. *bifurcaria* has been recorded in the Western Sahara (see [44]), but our field surveys failed to detect it beyond Tarfaya, which possibly already indicates a recent local shift. This edge population is large however, and does not show any external sign of stress. Nevertheless, this region was identified as a repository of genetic diversity of high conservation value and evolutionary potential, and as among the first to be negatively affected by ongoing climatic change.

The role of rear edges as important and vulnerable areas of diversification and endemism has long been recognized, particularly for terrestrial organisms but also for cold-temperate marine taxa; as our study shows, the same should apply to warm-temperate coastal species. Understudied low-latitude regions at the rear-edge of many species should thus receive added attention to allow range changes of warm-temperate species like *B*. *bifurcata* to be promptly detected, and their genetic consequences assessed.

## Supporting Information

S1 Fig Correlation matrix of environmental predictors used in Ecological Niche Modelling. The upper matrix shows the coefficient of correlation between predictors (sized by its numerical value), the lower matrix shows the scatter plot of pairs of predictors, and the diagonal shows the histogram and name of individual predictors.
(DOCX)

S2 Fig Number of genetic clusters of *Bifurcaria bifurcata* according to STRUCTURE. The “best” number (k, set from 1 to 12) was inspected using the choice criteria of Pritchard *et al*. (2000; left axis) and Evanno *et al*. (2005; right axis). Five iterations were run for each *K*.
(DOCX)

S1 Table Occurrence records used to build the Ecological Niche Model of *Bifurcaria bifurcata*. Coordinates (decimal degrees), countries, approximate locations, and original sources are reported for each site.
(DOCX)

S2 Table Sequences of primers and PCR conditions for the 6 microsatellite and the two plastid markers analyzed. (DOCX)

S3 Table Contribution of environmental variables to the Ecological Niche models of *Bifurcaria bifurcata*. Model accuracy was assessed using True Skill Statistics (TSS). Note the similarity between MARS and BRT models.
(DOCX)

S4 Table Pairwise differentiation of populations of *Bifurcaria bifurcata*. *F*_ST_ (θ) values are given above diagonal, in bold if non-significant. Jost’s *D*_est_ are given below diagonal. Population codes are as in Table 1.
(DOCX)
