# Supplementary material for: Genes Left Behind: Climate Change Threatens Cryptic Genetic Diversity in the Canopy-Forming Seaweed Bifurcaria bifurcata
Source: PLoS One. 2015 Jul 15;10(7):e0131530. doi: 10.1371/journal.pone.0131530 (PMC4503591; doi:10.1371/journal.pone.0131530)
Supplement: S1 Fig — The upper matrix shows the coefficient of correlation between predictors (sized by its numerical value), the lower matrix shows the scatter plot of pairs of predictors, and the diagonal shows the histogram and name of individual predictors. (DOCX) [file pone.0131530.s001.docx]

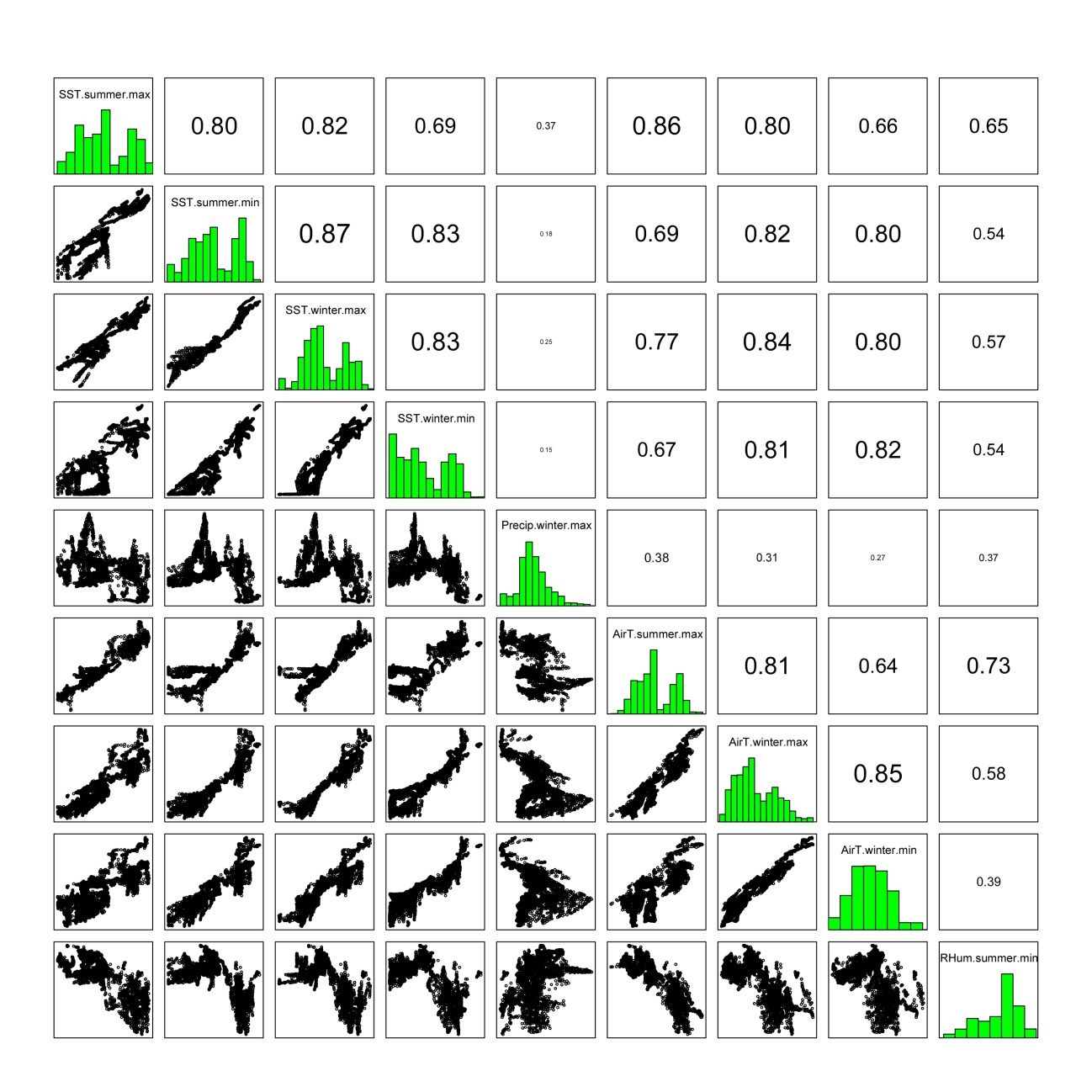


**S1 Fig. Correlation matrix of the environmental predictors used in ecological niche modelling.** The upper matrix shows the coefficient of correlation between predictors (sized by its numerical value), the lower matrix shows the scatter plot of pairs of predictors, and the diagonal shows the histogram and name of individual predictors.
