# Supplementary material for: Genes Left Behind: Climate Change Threatens Cryptic Genetic Diversity in the Canopy-Forming Seaweed Bifurcaria bifurcata
Source: PLoS One. 2015 Jul 15;10(7):e0131530. doi: 10.1371/journal.pone.0131530 (PMC4503591; doi:10.1371/journal.pone.0131530)
Supplement: S2 Fig — The “best” number (k, set from 1 to 12) was inspected using the choice criteria of Pritchard et al. (2000; left axis) and Evanno et al. (2005; right axis). Five iterations were run for each K. (DOCX) [file pone.0131530.s002.docx]

**
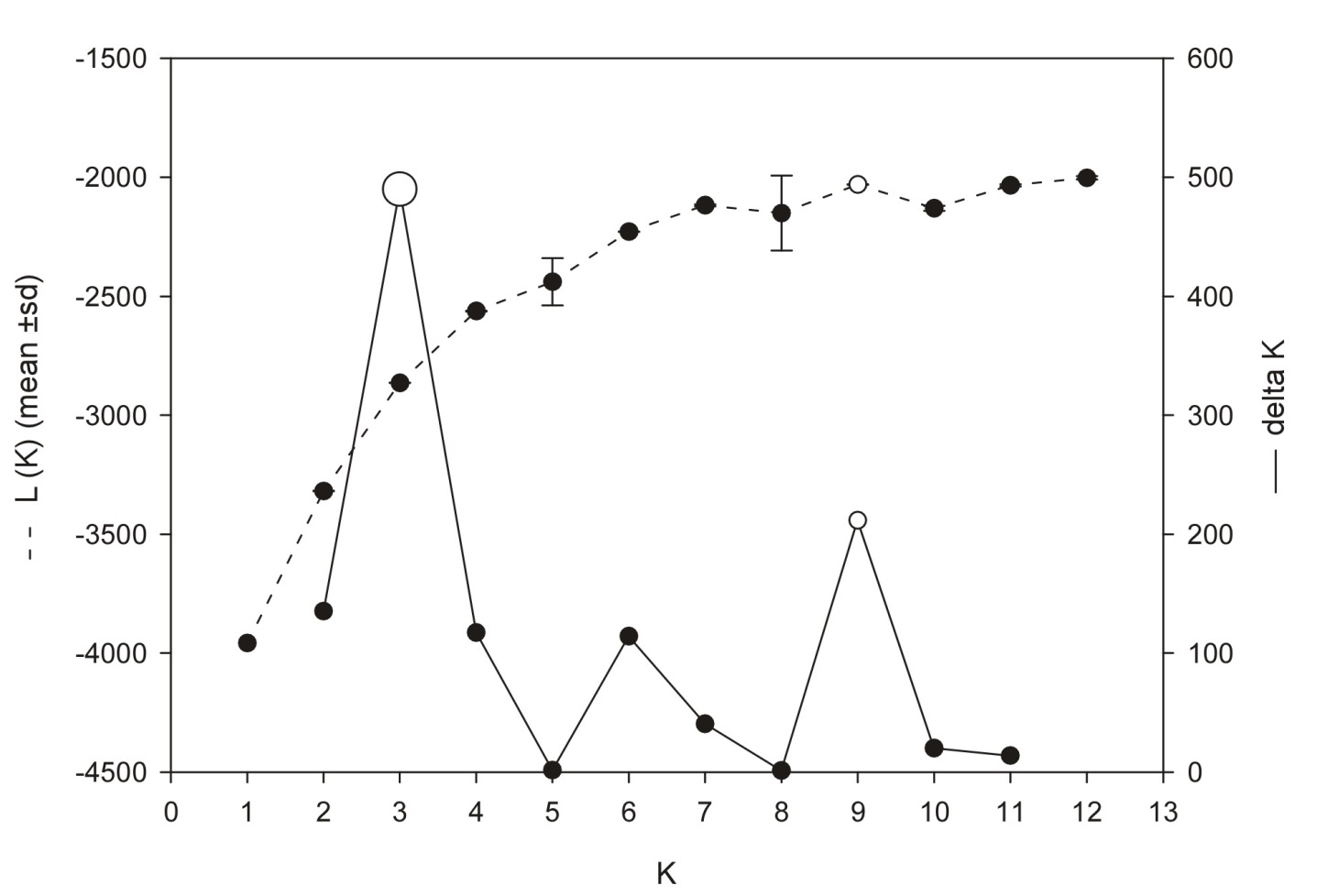
**

**S2 Fig. Number of genetic clusters of *Bifurcaria bifurcata* according to STRUCTURE.** The “best” number (k, set from 1 to 12) was inspected using the choice criteria of Pritchard *et al.* (2000; left axis) and Evanno *et al.* (2005; right axis). Five iterations were run for each *K*.
