# Supplementary material for: Genes Left Behind: Climate Change Threatens Cryptic Genetic Diversity in the Canopy-Forming Seaweed Bifurcaria bifurcata
Source: PLoS One. 2015 Jul 15;10(7):e0131530. doi: 10.1371/journal.pone.0131530 (PMC4503591; doi:10.1371/journal.pone.0131530)
Supplement: S1 Table — Coordinates (decimal degrees), countries, approximate locations, and original sources are reported for each site. (DOCX) [file pone.0131530.s003.docx]

**S1 Table. Occurrence records used to build the Ecological Niche Model of *Bifurcaria bifurcata*.** Coordinates (decimal degrees), countries, approximate locations, and original sources are reported for each site.

| **Latitude** | **Longitude** | **Country** | **Location** | **Source** |
| --- | --- | --- | --- | --- |
| 27.9176 | -12.9611 | Morocco | near Tarfaya | Sampled by the authors and/or colleagues |
| 28.4138 | -11.4024 | Morocco | near El Ouatia | Sampled by the authors and/or colleagues |
| 29.6325 | -10.0093 | Morocco | near Mirleft | <http://collections.si.edu/search/results.htm?tag.cstype=all&q=bifurcaria+bifurcata&fq=place:%22Morocco%22> |
| 30.6261 | -9.8849 | Morocco | Cap Ghir | S. Benhissoune. C.F. Boudouresque & M. Verlaque (2002) A checklist of the seaweeds of the Mediterranean and Atlantic coasts of Morocco. II. Phaeophyceae. Botanica Marina 45: 217-223 |
| 30.8380 | -9.8225 | Morocco | Imnsouane | <http://collections.si.edu/search/results.htm?tag.cstype=all&q=bifurcaria+bifurcata&fq=place:%22Morocco%22> |
| 31.0999 | -9.8323 | Morocco | Cap Tafelney | <http://collections.si.edu/search/results.htm?tag.cstype=all&q=bifurcaria+bifurcata&fq=place:%22Morocco%22> |
| 31.5111 | -9.7756 | Morocco | Essaouira | Sampled by the authors and/or colleagues |
| 31.9086 | -9.4642 | Morocco | Sidi Boulbra | Birje. J. ; Verlaque. M. ; Poydenot. F. (1996) Macrophytobenthos des platiers rocheux intertidaux et se mi-exposés de la région de Safi-Essaouira (Maroc occidental). Oceanologica Acta 19: 561-574 |
| 32.5438 | -9.2808 | Morocco | El Bdouza | Sampled by the authors and/or colleagues |
| 32.7499 | -9.0291 | Morocco | near Oualidia | Hellio. C.. Thomas-Guyon. H.. Culioli. G.. Piovetti. L.. Bourgougnon. N. and Le Gal. Y. (2001) Marine antifoulants from *Bifurcaria bifurcata* (Phaeophyceae. Cystoseiraceae) and other brown macroalgae. Biofouling. 17: 189-201. |
| 33.2063 | -8.5859 | Morocco | near El Jadida | Sampled by the authors and/or colleagues |
| 33.5317 | -7.8312 | Morocco | Dar Bouâaza | Ainane. T. (2011) Valorisation de la biomasse algale du Maroc: Potentialités pharmacologiques et Applications environnementales. cas des algues brunes Cystoseira tamariscifolia et Bifurcaria bifurcata. Thèse Présentée à la Faculté des Sciences Ben M’Sik pour obtenir le diplôme de DOCTORAT. Discipline: Chimie. Spécialité: Chimie Analytique. Faculté des Science Ben M’sik Casablanca. Université Hassan II – Casablanca |
| 33.8852 | -7.0229 | Morocco | Rose Marie. near Rabbat | Abboud. Y.; Saffaj. T.; Chagraoui. A.; Bouari. A.; Brouzi. K.; Tanane. O.; Ihssane. B. (2014) Biosynthesis. characterization and antimicrobial activity of copper oxide nanoparticles (CONPs) produced using brown alga extract (*Bifurcaria bifurcata*). Applied Nanoscience. 4: 571-576 |
| 37.2950 | -8.8693 | Portugal | Arrifana | Bárbara. I; Tapia. PD; Peteiro. C.. Berecibar. E.; Peña. V.; Sánchez. N.; Tavares. A.M.; Santos. R.; Secilla. A.. Fernández. P.R.; Bermejo. R.; García. V. (2012) Nuevas citas y aportaciones corologicas para la flora bentonica marina del atlantico de la peninsula iberica. Acta Bot. Malacitana. 37: 5-32. |
| 37.4470 | -8.7998 | Portugal | Odeceixe | Sampled by the authors and/or colleagues |
| 38.6899 | -9.3639 | Portugal | Cascais | Cabeçadas. G.; Monteiro. M.T.; Brogueira. M.J.; Guerra. M.; Gaudêncio. M.J.; Passos. M.; Cavaco. M.H.; Gonçalves. C.; Ferronha. H.; Nogueira. M.; Cabeçadas. P.; Oliveira. A.P.. (2004) Caracterização ambiental da zona costeira adjacente aos estuários do tejo e sado. Relatórios Científicos e Técnicos do IPIMAR. Série digital. nº 20). <https://www.ipma.pt/resources.www/docs/publicacoes.site/docweb/2004/Reln20final.pdf> |
| 38.7948 | -9.4917 | Portugal | Adraga | Pereira. S. G.; Lima. F. P.; Queiroz. N. C.; Ribeiro. P. A.; Santos. A. M. (2006) Biogeographic patterns of intertidal macroinvertebrates and their association with macroalgae distribution along the Portuguese coast. Hydrobiologia. 555: 185-192. |
| 38.9886 | -9.4208 | Portugal | Ribeira d'Ilhas | Sampled by the authors and/or colleagues |
| 39.1246 | -9.3914 | Portugal | Santa Cruz | Pereira. S. G.; Lima. F. P.; Queiroz. N. C.; Ribeiro. P. A.; Santos. A. M. (2006) Biogeographic patterns of intertidal macroinvertebrates and their association with macroalgae distribution along the Portuguese coast. Hydrobiologia. 555: 185-192. |
| 39.3765 | -9.3398 | Portugal | Baleal | <http://macoi.ci.uc.pt/spec_list_detail.php?spec_id=8&order=tpup> |
| 40.1777 | -8.9037 | Portugal | Buarcos | <http://macoi.ci.uc.pt/spec_list_detail.php?spec_id=8&order=tpup> |
| 41.0445 | -8.6525 | Portugal | Aguda | Pereira. S. G.; Lima. F. P.; Queiroz. N. C.; Ribeiro. P. A.; Santos. A. M. (2006) Biogeographic patterns of intertidal macroinvertebrates and their association with macroalgae distribution along the Portuguese coast. Hydrobiologia. 555: 185-192. |
| 41.2944 | -8.7368 | Portugal | Mindelo | Pereira. S. G.; Lima. F. P.; Queiroz. N. C.; Ribeiro. P. A.; Santos. A. M. (2006) Biogeographic patterns of intertidal macroinvertebrates and their association with macroalgae distribution along the Portuguese coast. Hydrobiologia. 555: 185-192. |
| 41.6962 | -8.8512 | Portugal | Viana | Sampled by the authors and/or colleagues |
| 41.8401 | -8.8751 | Portugal | Moledo | <http://macoi.ci.uc.pt/spec_list_detail.php?spec_id=8&order=tpup> |
| 42.2294 | -8.8972 | Spain | Ciés | Sibaja-Cordero. J.A. & Troncoso. J.S. (2011) Upper and lower limits of rocky shore organisms at different spatial scales and wave exposure (Islas Cíes. NW Spain). Thalassas. 27:81-100 |
| 43.0004 | -9.2611 | Spain | Lires | Sampled by the authors and/or colleagues |
| 43.3757 | -8.3393 | Spain | Coruña | Sampled by the authors and/or colleagues |
| 43.4542 | -8.3079 | Spain | Ferrol | Granja. A.; Cremades. J.; Bárbara. I. (1992) Catálogo de las algas bentónicas marinas de la Ría de Ferrol (Galicia. N.O. de la Península Ibérica) y consideraciones biogeográficas sobre su flora. Nova Acta Científica Compostelana (Bioloxía). 3:3-21 |
| 43.6496 | -8.0822 | Spain | Cedeira | Perez-Cirera. J. L.. 1975a. Catálogo floristico de las algas bentonicas de la Ria de Cedeira. NO de España. — An.Inst. bot. A. J. Cavanilles32. 53–74. |
| 43.5633 | -6.8738 | Spain | Porcia | Sampled by the authors and/or colleagues |
| 43.5520 | -6.5519 | Spain | Tourán | Morán (1994) Descripción de las comunidades dominadas por macroalgas en la costa de Asturias. Departamento de Biología de Organismos y Sistemas. Universidad de Oviedo. |
| 43.6126 | -5.7854 | Spain | Luanco | Fernández. C.. Niell. F.X. & Anadón. R. (1983). Comparación de dos comunidades de horizontes intermareales con abundancia de Bifurcaria bifurcata Ros. en las costas N y NO de España. Investigacion Pesquera 47(3): 435-455. |
| 43.5488 | -5.6416 | Spain | El Rinconín | Morán (1994) Descripción de las comunidades dominadas por macroalgas en la costa de Asturias. Departamento de Biología de Organismos y Sistemas. Universidad de Oviedo. |
| 43.5093 | -5.2707 | Spain | Lastres | Sampled by the authors and/or colleagues |
| 43.3963 | -4.6179 | Spain | Buelna | Morán (1994) Descripción de las comunidades dominadas por macroalgas en la costa de Asturias. Departamento de Biología de Organismos y Sistemas. Universidad de Oviedo. |
| 43.4700 | -3.7661 | Spain | Penísula de la Magdalena | <http://portal.ayto-santander.es/documentos/pgou/t01_informacion_urbanistica/informacion_urbanistica_9.pdf> |
| 43.4941 | -3.5211 | Spain | Noja | Sampled by the authors and/or colleagues |
| 43.3952 | -2.9880 | Spain | Meñakoz | Díez I.. Santolaria A.. Secilla A.. Gorostiaga J.M. (2009). Recovery stages over long-term monitoring of the intertidal vegetation in the ‘Abra de Bilbao’ area and on the adjacent coast (N. Spain). *European Journal of Phycology.* 44: 1-14. |
| 43.4459 | -2.7839 | Spain | Gaztelugatxe | Unknown (2006) Programa de vigilancia y control de la Introducción de especies invasoras en los ecosistemas litorales de la costa vasca. 2. Costa de Bizkaia. Departamiento de Medio Ambiente e Ordenacion del territorio del Gobierno Vasco. <http://www.ingurumena.ejgv.euskadi.net/r49-3074/eu/contenidos/informe_estudio/invasoras_costa/eu_doc/adjuntos/memoria2.pdf> |
| 43.3671 | -2.4967 | Spain | Leikitio |  |
| 43.2979 | -2.2725 | Spain | Zumaya | Sampled by the authors and/or colleagues |
| 46.6936 | -2.3088 | France | Yeu Sud | Barrilé *et al*. (2007) Mise en place de la DCE dans les masses d'eau côtières des Pays de la Loire - Prospection de la flore et de la faune benthiques et proposition d'un réseau de surveillance. IFREMER. <http://archimer.ifremer.fr/doc/00000/2591/> |
| 46.7295 | -2.3601 | France | Yeu Nord |  |
| 47.3794 | -2.5518 | France | Piriac-sur-Mer | Sampled by the authors and/or colleagues |
| 47.4734 | -3.1112 | France | Quiberon | Hellio. C.. Thomas-Guyon. H.. Culioli. G.. Piovetti. L.. Bourgougnon. N. and Le Gal. Y. (2001) Marine antifoulants from *Bifurcaria bifurcata* (Phaeophyceae. Cystoseiraceae) and other brown macroalgae. Biofouling. 17: 189-201 |
| 47.8170 | -4.3823 | France | Saint Guénolé | Klervi Le Lann. K.; Rumin. J.; Cérantola. S.; Culioli. G.; Stiger-Pouvreau. V. (2013) Spatiotemporal variations of diterpene production in the brown macroalga *Bifurcaria* bifurcata from the western coasts of *Brittany* (France). Journal of Applied Phycology. 26: 1207-1214. |
| 48.3543 | -4.5587 | France | Plouzané | Le Lann. K.. Jégou. C. and Stiger-Pouvreau. V. (2008) Effect of different conditioning treatments on total phenolic content and antioxidant activities in two Sargassacean species: Comparison of the frondose Sargassum muticum (Yendo) Fensholt and the cylindrical Bifurcaria bifurcata R. Ross. Phycological Research. 56: 238–245 |
| 48.4035 | -4.9459 | France | Ile de Molene | <http://inpn.mnhn.fr/zone/znieff/espece/72522> |
| 48.5590 | -4.7058 | France | Port Sall | Hellio. C.. Thomas-Guyon. H.. Culioli. G.. Piovetti. L.. Bourgougnon. N. and Le Gal. Y. (2001) Marine antifoulants from *Bifurcaria bifurcata* (Phaeophyceae. Cystoseiraceae) and other brown macroalgae. Biofouling. 17: 189-201. |
| 48.6632 | -4.2164 | France | Porsmeur | Klervi Le Lann. K.; Rumin. J.; Cérantola. S.; Culioli. G.; Stiger-Pouvreau. V. (2013) Spatiotemporal variations of diterpene production in the brown macroalga *Bifurcaria* bifurcata from the western coasts of *Brittany* (France). Journal of Applied Phycology. 26: 1207-1214. |
| 48.7290 | -3.9904 | France | Roscoff | Sampled by the authors and/or colleagues |
| 48.7190 | -3.8195 | France | Pointe de Primel | Perez René. Audouin Jacques. Braud Jean-Paul. Uhm K.B (1973). Répartition des grands champs d'algues brunes sur les côtes françaises de la Manche Occidentale entre l'Ile grande et l'Ile de Siec. Science et Pêche. 226. 1-12. Open Access version : <http://archimer.ifremer.fr/doc/00000/7092/> |
| 48.8908 | -3.4657 | France | Malban | Gall *et al.* (2007) Lieux de surveillance du Benthos. Région Bretagne. Suivi stationnel des roches intertidales (Flore). IFREMER  <http://www.rebent.org//medias/documents/www/contenu/documents/REBENT_RAF_035_SyntheseSuiviStationnel_ed2011_v2r0.pdf> |
| 48.8407 | -2.9865 | France | l’île de Bréhat |  |
| 48.6351 | -2.1386 | France | Saint-Briac | Sampled by the authors and/or colleagues |
| 48.8308 | -1.5895 | France | Granville | <http://inpn.mnhn.fr/zone/znieff/espece/72522> |
| 48.8762 | -1.8421 | France | Chausey |  |
| 49.4743 | -2.6040 | UK | Guernsey | Crisp D.J. & Southward A.J. (1958). The Distribution of Intertidal Organisms Along the Coasts of the English Channel. Journal of the Marine Biological Association of the United Kingdom. 37:157-203. |
| 49.7295 | -2.1828 | UK | Alderney |  |
| 49.6567 | -1.8736 | France | Herqueville | <http://inpn.mnhn.fr/zone/znieff/espece/72522> |
| 49.7098 | -1.8901 | France | Anse Saint Martin |  |
| 49.6973 | -1.4728 | France | Cap Levi | Vonthron-Sénécheau. C. Kaiser. M. Devambez. I. Vastel. A. Mussio. I. Rusig. A-M. (2011) Antiprotozoal activities of organic extracts from french marine seaweeds. Marine Drugs. 9: 922–933 |
| 49.6954 | -1.2742 | France | Pointe de Barfleur | <http://inpn.mnhn.fr/zone/znieff/espece/72522> |
| 49.5895 | -1.2388 | France | Ile de Tatihou |  |
| 50.5137 | -2.4581 | UK | Portland Bill | Mieszkowska. N.. Kendall. M.A.. Hawkins. S.J.. Leaper. R.. Williamson. P.. Hardman-Mountford. N.J. and Southward. A.J. (2006) Changes in the Range of Some Common Rocky Shore Species in Britain – A Response to Climate Change? Hydrobiologia. 555. (1). 241-251 |
| 50.3416 | -3.5653 | UK | Darthmouth |  |
| 50.3165 | -4.0846 | UK | Wenbury | Sampled by the authors and/or colleagues |
| 50.3440 | -4.4526 | UK | Looe | Martins GM. Hawkins SJ. Thompson RC. Jenkins SR (2007) Community structure and functioning in intertidal rockpools: effects of pool size and shore height at different successional stages Marine Ecology Progress Series 329:43-55. |
| 50.0836 | -5.5374 | UK | Mouse Hole | Sampled by the authors and/or colleagues |
| 50.2076 | -5.4719 | UK | St. Ives | Sampled by the authors and/or colleagues |
| 50.5738 | -4.9241 | UK | Polzeath | Sampled by the authors and/or colleagues |
| 50.7406 | -4.6404 | UK | Crackington Haven | <http://www.jncc.gov.uk/marine/biotopes/biotope_image.aspx?biotope=JNCCMNCR00001160&cd=3548&image=IMG0079.JPG> |
| 51.1255 | -4.2439 | UK | Croyde Beach | <http://www.marlin.ac.uk/sightingsMapper.php?sps=Bifurcaria%20bifurcata> |
| 51.6356 | -5.0546 | UK | Castlemartin Range | Bunker. F. P. O. & Bunker A. (1998) Biotope studies on selected rocky shores of South Pembrokeshire following the Sea Empress oil spill. CCW SEA EMPRESS CONTRACT REPORT DISTRIBUTION. |
| 51.7311 | -5.2457 | UK | Renny Slip Bay | Jones. W.E. & Williams R. The Seaweeds of Dale. |
| 51.5911 | -8.7999 | Ireland | Clonakilty | Sampled by the authors and/or colleagues |
| 51.6797 | -9.4620 | Ireland | Bantry Bay | Guiry. M. D.. "The Marine Algal Flora of Bantry Bay. Co. Cork". Irish Fisheries Investigations Series B. Department of Agriculture and Fisheries (Fisheries Division) 1973 |
| 52.6847 | -9.6517 | Ireland | Kilkee | <http://www.algaebase.org/search/species/detail/?species_id=Cf11899ab0bf74ef1&-session=abv4:C2D2FB15187da25002WLU1983135> |
| 52.8453 | -9.4408 | Ireland | Spanish Point | <http://www.algaebase.org/search/species/detail/?species_id=n3beb1af3396aad0e&sk=10> |
| 53.1413 | -9.2249 | Ireland | Galway | Sampled by the authors and/or colleagues |
| 53.1101 | -9.6632 | Ireland | Aran Islands | De Valéra. M. (1962). Some aspects of the problem of the distribution of *Bifurcaria bifurcata* (Velley) Ross on the shores of Ireland. north of the Shannon Estuary. *Proceedings of the Royal Irish Academy* 62B: 77-101. |
| 53.6133 | -10.2257 | Ireland | Inishbofin |  |
| 54.0963 | -10.1248 | Ireland | Cloughanakilla |  |
| 54.2930 | -8.9691 | Ireland | Easky |  |
| 54.6638 | -8.7768 | Ireland | Malin beg |  |
| 55.2464 | -7.7567 | Ireland | Ballyhoorisky Point | Unknown (2005) Conservation Plan for Ballyhoorisky Point to Fanad Head cSAC — Site Code 1975. Dept. of Environment. Heritage and Local Government. <http://www.npws.ie/publications/archive/CP001975.pdf> |
