# Supplementary material for: Genes Left Behind: Climate Change Threatens Cryptic Genetic Diversity in the Canopy-Forming Seaweed Bifurcaria bifurcata
Source: PLoS One. 2015 Jul 15;10(7):e0131530. doi: 10.1371/journal.pone.0131530 (PMC4503591; doi:10.1371/journal.pone.0131530)
Supplement: S2 Table — (DOCX) [file pone.0131530.s004.docx]

**S2 Table. Sequences of primers and PCR conditions for the 6 microsatellite and the two plastid markers analysed.**

| *Locus* | *Primers 5’ 🡪 3’* | *Mg (mM)* | *Cycles (n)* | *T_a_ (*º*C)* |
| --- | --- | --- | --- | --- |
| Bb05 | F*: TGCCCGGCAAAGAAGAGGTG  R: CGTACGCTGGAACGCAAGGT | 2 | 35 | 60 |
| Bb07 | F*: CGGATTTCGGCTCGTTGGGT  R: AGCCTAATCCCAGCTTCTTGGG | 2 | 35 | 60 |
| Bb12 | F*: CGCATCATGAACCCAAGCACG  R: TTTGGCTGCGAGCGAGACTG | 2 | 35 | 60 |
| nBb10 | F*: TTGACTCCGCCATTCGTAGG  R: TCTAAATTAAATTTCGAGTTATTAGC | 2 | 35 | 60 |
| nBb13 | F*: CGGACCATAGTGGCTTATGC  R: GCAATATATCAAAATGTGGAGAGG | 2 | 35 | 60 |
| nBb19 | F*CGCCGATATTGACTCGATG  R: CGAACACGTAACCACACGC | 2 | 35 | 60 |
| mt*cox3* | F: CCATGGCCTTTTGTGGCTGCCT  R: GCCGGGGCTTTAAACCCGGAAT | 2 | 30 | 62 |
| cp*rbc* | F: GGATGCGTATGTCAGGTGTAGA  R: TGAAAAACATCCTTGTGTAAGTCTCA | 2 | 30 | 60 |

* labelled (NED, FAM, HEX, ROX) primers.
