# Supplementary material for: Genes Left Behind: Climate Change Threatens Cryptic Genetic Diversity in the Canopy-Forming Seaweed Bifurcaria bifurcata
Source: PLoS One. 2015 Jul 15;10(7):e0131530. doi: 10.1371/journal.pone.0131530 (PMC4503591; doi:10.1371/journal.pone.0131530)
Supplement: S3 Table — Model accuracy was assessed using True Skill Statistics (TSS). Note the similarity between MARS and BRT models. (DOCX) [file pone.0131530.s005.docx]

**
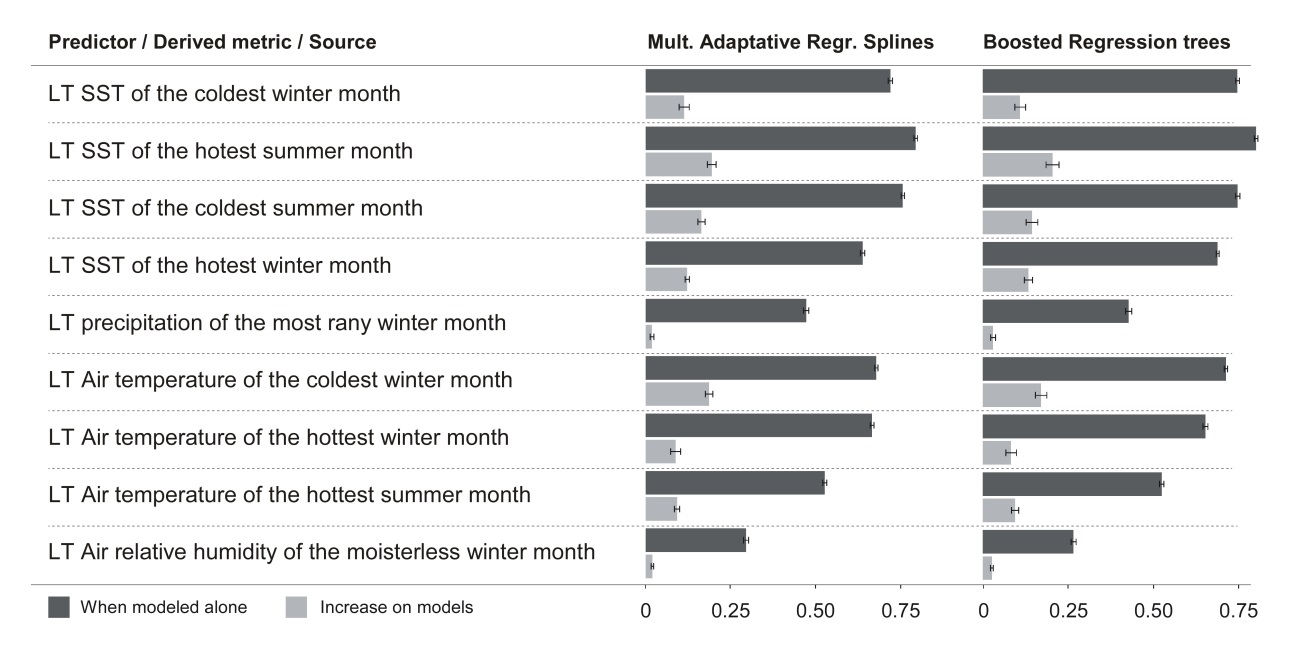
S3 Table. Contribution of environmental variables to the Ecological Niche Models of *Bifurcaria bifurcata*.** Model accuracy was assessed using True Skill Statistics (TSS). Note the similarity between MARS and BRT models.
