# Supplementary material for: Genes Left Behind: Climate Change Threatens Cryptic Genetic Diversity in the Canopy-Forming Seaweed Bifurcaria bifurcata
Source: PLoS One. 2015 Jul 15;10(7):e0131530. doi: 10.1371/journal.pone.0131530 (PMC4503591; doi:10.1371/journal.pone.0131530)
Supplement: S4 Table — F ST (θ) values are given above diagonal, in bold if non-significant. Jost’s D est are given below diagonal. Population codes are as in Table 1. (DOCX) [file pone.0131530.s006.docx]

**S4 Table**. **Pairwise differentiation of populations of *Bifurcaria bifurcata*.** *F*_ST_ (θ) values are given above diagonal, in bold if non-significant. Jost’s *D*_est_ are given below diagonal. Population codes are as in Table 2.

|  | **TAR** | **OUA** | **ESS** | **BDO** | **JAD** | **ODE** | **ERI** | **VIA** | **LIR** | **RCO** | **POR** | **LAS** | **ZUM** | **PSM** | **STM** | **PLY** | **COR** | **GAL** |
| --- | --- | --- | --- | --- | --- | --- | --- | --- | --- | --- | --- | --- | --- | --- | --- | --- | --- | --- |
| **TAR** | -- | 0,145 | 0,486 | 0,486 | 0,413 | 0,398 | 0,429 | 0,521 | 0,572 | 0,494 | 0,551 | 0,386 | 0,584 | 0,500 | 0,651 | 0,580 | 0,645 | 0,558 |
| **OUA** | 0,061 | -- | 0,566 | 0,571 | 0,490 | 0,518 | 0,549 | 0,589 | 0,653 | 0,560 | 0,637 | 0,499 | 0,693 | 0,562 | 0,742 | 0,662 | 0,699 | 0,671 |
| **ESS** | 0,404 | 0,483 | -- | 0,411 | 0,311 | 0,435 | 0,400 | 0,418 | 0,498 | 0,495 | 0,216 | 0,241 | 0,627 | 0,250 | 0,580 | 0,498 | 0,476 | 0,295 |
| **BDO** | 0,330 | 0,387 | 0,112 | -- | 0,247 | 0,519 | 0,503 | 0,582 | 0,616 | 0,588 | 0,578 | 0,446 | 0,724 | 0,502 | 0,687 | 0,616 | 0,646 | 0,621 |
| **JAD** | 0,310 | 0,356 | 0,113 | 0,061 | -- | 0,317 | 0,289 | 0,345 | 0,372 | 0,385 | 0,351 | 0,222 | 0,631 | 0,220 | 0,468 | 0,375 | 0,472 | 0,415 |
| **ODE** | 0,207 | 0,177 | 0,137 | 0,163 | 0,077 | -- | **0,037** | 0,200 | 0,243 | 0,137 | 0,411 | 0,125 | 0,597 | 0,369 | 0,527 | 0,279 | 0,662 | 0,485 |
| **ERI** | 0,245 | 0,258 | 0,122 | 0,162 | 0,073 | 0,003 | -- | 0,222 | 0,141 | 0,231 | 0,358 | 0,133 | 0,631 | 0,337 | 0,337 | 0,137 | 0,617 | 0,441 |
| **VIA** | 0,263 | 0,223 | 0,122 | 0,166 | 0,065 | 0,018 | 0,023 | -- | 0,204 | 0,087 | 0,296 | 0,206 | 0,752 | 0,354 | 0,609 | 0,286 | 0,731 | 0,485 |
| **LIR** | 0,283 | 0,294 | 0,124 | 0,167 | 0,069 | 0,013 | 0,004 | 0,009 | -- | 0,273 | 0,443 | 0,325 | 0,830 | 0,463 | 0,413 | **-0,005** | 0,752 | 0,638 |
| **RCO** | 0,246 | 0,208 | 0,122 | 0,164 | 0,061 | 0,013 | 0,017 | 0,005 | 0,013 | -- | 0,450 | 0,262 | 0,709 | 0,453 | 0,658 | 0,360 | 0,745 | 0,600 |
| **POR** | 0,343 | 0,399 | 0,033 | 0,177 | 0,072 | 0,051 | 0,037 | 0,020 | 0,029 | 0,029 | -- | 0,192 | 0,737 | 0,254 | 0,613 | 0,453 | 0,639 | 0,194 |
| **LAS** | 0,232 | 0,195 | 0,089 | 0,155 | 0,059 | 0,014 | 0,018 | 0,026 | 0,048 | 0,037 | 0,028 | -- | 0,537 | 0,133 | 0,512 | 0,342 | 0,525 | 0,208 |
| **ZUM** | 0,270 | 0,286 | 0,189 | 0,306 | 0,228 | 0,060 | 0,111 | 0,104 | 0,136 | 0,083 | 0,154 | 0,105 | -- | 0,736 | 0,929 | 0,833 | 0,769 | 0,845 |
| **PSM** | 0,307 | 0,305 | 0,061 | 0,146 | 0,025 | 0,063 | 0,059 | 0,044 | 0,057 | 0,057 | 0,019 | 0,022 | 0,194 | -- | 0,605 | 0,467 | 0,541 | 0,266 |
| **STM** | 0,324 | 0,411 | 0,124 | 0,167 | 0,069 | 0,044 | 0,023 | 0,025 | 0,008 | 0,029 | 0,033 | 0,060 | 0,212 | 0,061 | -- | 0,257 | 0,807 | 0,782 |
| **PLY** | 0,298 | 0,336 | 0,123 | 0,167 | 0,069 | 0,024 | 0,005 | 0,013 | 0,000 | 0,017 | 0,030 | 0,051 | 0,161 | 0,057 | 0,004 | -- | 0,740 | 0,633 |
| **COR** | 0,533 | 0,554 | 0,150 | 0,284 | 0,138 | 0,189 | 0,138 | 0,204 | 0,151 | 0,221 | 0,135 | 0,153 | 0,097 | 0,085 | 0,112 | 0,129 | -- | 0,754 |
| **GAL** | 0,267 | 0,334 | 0,054 | 0,164 | 0,073 | 0,024 | 0,022 | 0,029 | 0,040 | 0,030 | 0,015 | 0,016 | 0,160 | 0,018 | 0,040 | 0,039 | 0,170 | -- |
